# Microfabricated Organ-Specific Models of Tumor Microenvironments

**DOI:** 10.1146/annurev-bioeng-110222-103522

**Published:** 2025-05

**Authors:** Jeong Min Oh, Yongkuk Park, Jungwoo Lee, Keyue Shen

**Affiliations:** 1 Alfred E. Mann Department of Biomedical Engineering, University of Southern California, Los Angeles, California, USA; 2 Department of Chemical Engineering, University of Massachusetts, Amherst, Massachusetts, USA; 3 Department of Biomedical Engineering, University of Massachusetts, Amherst, Massachusetts, USA; 4 Molecular and Cellular Biology Graduate Program, University of Massachusetts, Amherst, Massachusetts, USA; 5 Norris Comprehensive Cancer Center, University of Southern California, Los Angeles, California, USA

**Keywords:** carcinogenesis, tumor microenvironment, TME, metastasis, microfabricated model, microphysiological systems, MPS

## Abstract

Despite the advances in detection, diagnosis, and treatments, cancer remains a lethal disease, claiming the lives of more than 600,000 people in the United States alone in 2024. To accelerate the development of new therapeutic strategies with improved responses, significant efforts have been made to develop microfabricated in vitro models of tumor microenvironments (TMEs) that address the limitations of animal-based cancer models. These models incorporate several advanced tissue engineering techniques to better reflect the organ- and patient-specific TMEs. Additionally, microfabricated models integrated with next-generation single-cell omics technologies provide unprecedented insights into patient’s cellular and molecular heterogeneity and complexity. This review provides an overview of the recent understanding of cancer development and outlines the key TME elements that can be captured in microfabricated models to enhance their physiological relevance. We highlight the recent advances in microfabricated cancer models that reflect the unique characteristics of their organs of origin or sites of dissemination.

## 1. INTRODUCTION

Cancer, “a disease in which some of the body’s cells grow uncontrollably and spread to other parts of the body” (as defined by the National Cancer Institute; https://www.cancer.gov/about-cancer/understanding/what-is-cancer), is the second leading cause of death worldwide. Over the past decade, the frustratingly low US Food and Drug Administration (FDA) approval rate of anticancer drugs (5.3%) after substantial time and financial investment has fueled the pursuit of advanced cancer models with improved preclinical testing outcomes (1). These models are essential tools that enable complex biological studies, facilitate new therapy development, and improve therapy responses. To bridge the translational gap between preclinical and clinical outcomes more robustly and sustainably, there is a growing interest in finding alternatives to animal-based cancer models. Notably, the interest in reducing animal testing is underscored by the FDA Modernization Act 2.0, which was recently signed into law on December 29, 2022 (2). Advances in tissue engineering have propelled in vitro cancer models to capture the cellular and molecular processes of the cancer microenvironment in a highly organized and scalable manner (3). These models incorporate advanced fabrication methods, patient-derived cells and extracellular matrices, mechanical stimuli, imaging, and multi-omics techniques to recapitulate the heterogeneity of various organ- and patient-specific cancers. In this review, we first outline the current understanding of cancer development and identify the key elements of the tumor microenvironment (TME), which serve as the design principles for physiologically relevant cancer models. Next, we highlight recently developed primary tumor models of the lung, liver, brain, and bone that reflect the key anatomy and physiology of their organs of origin. These organ-specific microfabricated cancer models are promising candidates for reducing and potentially replacing animal-based cancer models, either partially or fully, in the future.

## 2. MICROENVIRONMENTAL CONTEXTS OF CANCER PROGRESSION

The development of microfabricated cancer models builds upon and adds to our understanding of cancer progression, which has evolved from a traditional, cancer cell–centric view to a more comprehensive perspective, where cancer is largely driven by the dynamic interplay between cancer cells and their microenvironments. Tumor initiation is significantly influenced by the balance of the immune system, as it both promotes and constrains cancer development (4). In the early stages of tumor development, cytotoxic immune cells, such as natural killer (NK) cells and CD8^+^ T cells, may recognize and generate antitumor responses that eliminate cancer cells at the primary tumor sites (5, 6). However, immune surveillance for cancer cells may fail due to mechanisms involving inflammation and loss of antigenicity and/or immunogenicity (7). Immune evasion is further sustained by other immune cells, such as regulatory dendritic cells (8), regulatory T cells (9), myeloid-derived suppressor cells (MDSCs) (10), and tumor-associated macrophages (TAMs) (4).

The surviving cancer cells continue to modify their TME by recruiting and reprogramming noncancerous cells (e.g., fibroblasts, endothelial cells, and immune cells) and reshaping noncellular components [e.g., extracellular matrix (ECM)] (11, 12). As tumors grow, the rate of oxygen consumption outpaces the supply, leading to the formation of hypoxia in the TME (13). Tumor hypoxia is a prominent driver of signal activation and cellular reprogramming (14). Hypoxia stimulates various inflammatory and profibrotic growth factors, such as transforming growth factor β (TGF-β), which activate fibroblasts and induce their differentiation into cancer-associated fibroblasts (CAFs) (15). CAFs promote tumor ECM remodeling, including new ECM deposition, degradation, and cross-linking (16). Vascular endothelial cells are activated by the upregulated vascular endothelial growth factor (VEGF), which stimulates excessive angiogenesis, resulting in leaky and disorganized vascular formation that further complicates the nutrient and oxygen availability in the TME (17).

Malignant tumors expand beyond the primary tumor sites by local invasion and distant metastasis (18). To expand to the surrounding tissue, epithelial cancer cells must breach the basement membrane, which serves as a physical barrier between the epithelium and the surrounding stroma. Cancer cells and cancer-associated stromal cells (e.g., CAFs) produce degradative enzymes that disrupt the architecture and mechanical properties of the basement membrane (18). CAFs also exert physical forces directly on the ECM network, which generates gaps and alignments that support cancer invasion (19). During this process, the vascular basement membrane and the endothelial barrier may be disrupted (20), allowing tumor cells to invade and enter the circulation (either blood or lymphatic), known as intravasation in the metastatic cascade (21). Notably, other TME elements, such as TAMs, have been observed to promote intravasation (22–25). TAMs secrete proteolytic enzymes that further break down the ECM around the vessels and secrete epidermal growth factors to attract tumor cells (26). The occurrence of intravasation is remarkably frequent, with approximately 1–4 × 10^6^ cells per gram of tumor cells entering the vasculature daily (24).

Upon entering the circulation, tumor cells are subjected to multiple survival barriers, including physical stress and immune surveillance (27, 28). It is estimated that much less than 0.01% of the tumor cells successfully colonize the secondary sites. Tumor cells enter the circulation as single circulating tumor cells (CTCs) or in clusters, and CTC clusters have a 23- to 50-fold increased metastatic potential compared with the single CTCs (29). CTC clusters are composed of heterogeneous cell types that originate from the primary TME, including cancer cells, stromal cells, and immune cells, and each cell type contributes to the survival of the cluster (30). For example, neutrophils within the cluster suppress leukocyte activation (31), while platelets protect tumor cells from shear stress and NK-mediated cell lysis (32). Transcription factors associated with stemness and proliferation, including OCT4, NANOG, SOX2, and SIN3A, are also hypomethylated in CTC clusters (33).

Surviving CTCs colonize distant tissue sites through extravasation, which is a complex process involving adhesion molecules (e.g., integrins), chemokines (e.g., CXCL12), and cells of the premetastatic microenvironment (34). Generally, CTCs bind coagulation factors and arrest in capillaries by size-dependent restriction (35). Entrapped CTCs may exit vasculature by proliferating and rupturing the capillary, by transendothelial migration (27, 36), or by inducing necroptosis of endothelial cells (37). Disseminated tumor cells (DTCs) often lie dormant for years and even decades until their surrounding microenvironment at the secondary site becomes favorable for their regrowth (38), whereas their distribution is organ specific and highly dependent on the cross talk between the primary TME and the secondary sites (39–41). For example, capillaries of the liver and the bone marrow are lined with highly permeable fenestrated endothelial cells, while those of the brain are tightly regulated and wrapped by pericytes, encapsulated in a double-layered basement membrane, and further surrounded by astrocytes (42). Consistent with this observation, bone is the most common site of metastasis across all tumor types (24, 43).

## 3. ELEMENTS OF TMEs IN MICROFABRICATED MODELS

Based on these advances in the understanding of cancer progression, significant progress has been made in creating microfabricated models of cancer microenvironments to recapitulate and reveal the biology of cancer as well as to evaluate new therapeutic interventions. These models integrate microscale engineering principles originally developed for microelectronics, such as photolithography, with new human-cell/stem-cell sources, natural/synthetic biomaterials, and tissue engineering principles, which have revolutionized cell and tissue culture systems (44). Fundamentally, microfabricated models have a compartment(s) designated for cell culture (2-D monolayer or 3-D cell-laden ECM) and a microfluidic channel(s) connected to the cell culture compartment (45). Microfluidic technology enables precise patterning of various cells within models to mimic complex physiological structures, as well as controlled delivery of environmental factors such as nutrients, waste products, and chemokines. Various fabrication methods (e.g., soft lithography, injection molding, 3D printing) and materials (e.g., glass, silicone, plastic) are utilized to generate microfluidic-based platforms (46). The history and the biomedical application of microfluidics are further described by Albert Folch (47).

**Figure 1:**
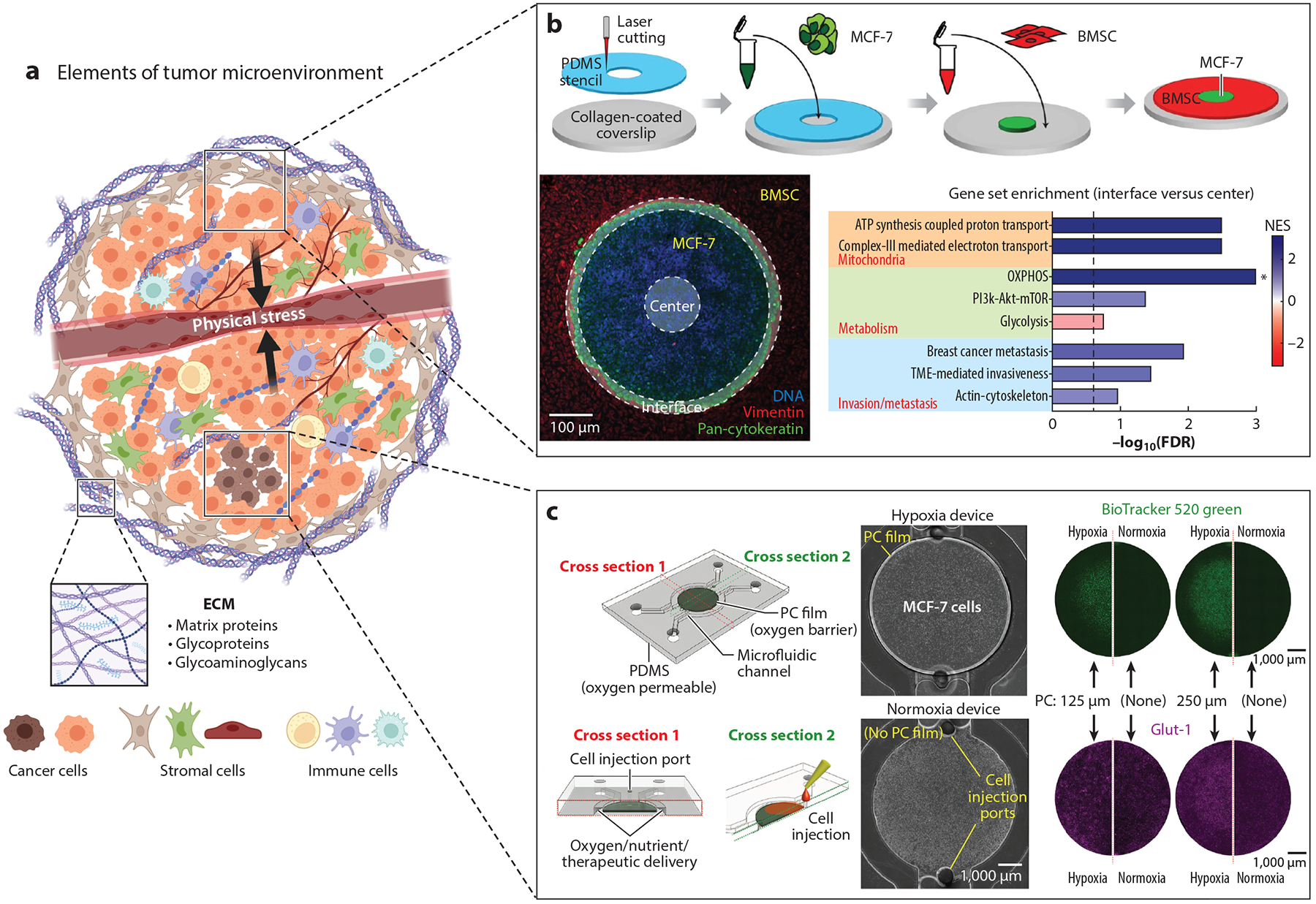
Elements of the TME and microfabricated breast TME models. (*a*) The TME is a complex landscape composed of multiple elements: cells, ECM, geometrical constraints, and physical stresses. Panel adapted from images created in BioRender; Oh, J. 2025. https://BioRender.com/m78b386. (*b*) Microfabricated model of breast tumor-stroma interactions. Panel adapted from Reference 48 (CC BY 4.0) (*c*) Microfabricated model of breast tumor hypoxia. Panel adapted with permission from Reference 49; copyright 2022 American Chemical Society. Abbreviations: BMSC, bone marrow stromal cell; ECM, extracellular matrix; FDR, false discovery rate; NES, normalized enrichment score; OXPHOS, oxidative phosphorylation; PC, polycarbonate; PDMS, polydimethylsiloxane; TME, tumor microenvironment.

In this review, the term microfabricated model refers to all engineered cell/tissue culture platforms that enable the control of the cell/tissue microenvironment with high spatiotemporal precision. The specific designs of the microfabricated models vary greatly depending on the specific disease contexts and organ/tissue sites. Generally, researchers need to find a balance between the complexity necessary for recapitulating the physiological characteristics and the overall throughput and cost-effectiveness needed for scientific and medical discoveries. Capturing the complex landscape of cancer progression requires combining multiple components of the TME within the microfabricated models. The TME elements can be broadly organized into the following: cells, ECM, geometrical constraints, and physical stresses (Figure 1*a*). This section briefly overviews each TME element and highlights the resources or surrogates often used in microfabricated models.

### 3.1. Cells

The cellular composition of the TME varies between tumor types. It features diverse cell populations, including noncancerous parenchymal cells, cancer cells, immune cells, fibroblasts, endothelial cells, adipocytes, and other tissue-resident cell types (50, 51). Numerous factors determine the functional states of these cells, including tissue sites, disease stages, and patient characteristics (52, 53). Therefore, it is critical for the microfabricated models to include relevant cells in such contexts. Multicellular tumor models (e.g., primary stromal cells and tumor cells) require optimization of their coculture conditions (54). Several key factors, such as initial seeding cell number/ratios and media formulations, are crucial for enhancing the relevance of cell–cell interactions and maintaining cellular heterogeneity. Sources of tumor cells include normal and cancer cell lines, (onco-)genetically modified stem cells, or patient-derived primary cells. Cell lines have served as an indispensable resource in establishing TME models (55–57). However, cell lines are established absent of other cell types; they also have limited functional capacity or heterogeneity as observed in patients. Patient-derived primary cells maximally preserve the (patho-)physiological cellular states in the TME. However, the availability of primary cells and their ex vivo expansion are limited. Maintaining in vivo phenotypes and processes of primary cells in microfabricated models remains a critical challenge. The pros and cons must be carefully considered, and a combination of cell sources may be necessary for the optimal development of the microfabricated model.

### 3.2. Extracellular Matrix

The ECM comprises up to 60% of the tumor mass and interacts with all cell types in the TME (58). The various ECM molecules found in the TME include matrix proteins (e.g., collagen and elastin), glycoproteins (e.g., fibronectin and laminin), and glycosaminoglycans (e.g., heparin/heparan sulfate and hyaluronic acid) (59). The exact compositions depend on the organs of origin and disease stages (15, 60). For example, in invasive ductal carcinomas, collagen production shifts toward collagen type I and collagen type III compared with benign mammary lesions (60). Changes in ECM composition can alter its biochemical and mechanical properties. The mechanical properties of the ECM, including stiffness and viscoelasticity, influence cancer development, including proliferation, metastasis, therapeutic resistance, and immunosuppression (61). Various ECM components used in microfabricated TME models include synthetic materials [e.g., polyethylene glycol and poly(lactic-co-glycolic) acid (PLGA)] and natural biomaterials from tissues (e.g., collagen and fibrin), as summarized in several reviews (59, 62, 63). Generally, natural ECMs exhibit higher biocompatibility and biodegradability than synthetic polymers (64). Nevertheless, synthetic polymers offer high reproducibility (i.e., low batch-to-batch variability) and tunability (i.e., biophysical properties) that address the limitation of natural ECMs (65). Matrigel^®^, a basement-membrane matrix from Engelbreth-Holm-Swarm mouse sarcoma, is widely used in microfabricated models involving tumor organoids. Such tumor-derived ECMs contain biologically active, TME-relevant components. However, high batch-to-batch variations may compromise the reproducibility of the microfabricated models.

### 3.3. Geometrical Constraints

Geometrical constraints pertain to the physical structures and spatial arrangements that impact cellular behaviors and interactions within the TME. For example, markers of epithelial-mesenchymal transition are preferentially expressed in cancer cells near the tumor–stroma interface (66). A micropatterned tumor model was developed to show that interfacial interactions promote a gene expression signature correlated with malignant progression, which resembles patient tumors and can be reversed by drug treatment (67). In a separate study, this model was used to reveal that the spatial constraint imposed by the tumor–stromal interface differentially regulates mitochondrial and metabolic activities in the tumor (48) (Figure 1*b*). Heterogeneous growth and spatial arrangement of cancer cells impact their accessibility to the vasculature. As a result, gradients of microenvironmental factors (e.g., oxygen, nutrients, pH, and soluble factors) are observed in the TME (68). Microfabricated models can recapitulate such impact under geometrical constraints of the TME (49, 69, 70). For example, Oh et al. (49) developed a scalable microdevice to naturally induce hypoxia/metabolic gradients by integrating oxygen diffusion barriers onto a layer of oxygen-consuming cancer cells, which recapitulated tumor hypoxia in both 2D and 3D arrangement without external oxygen control (Figure 1*c*).

### 3.4. Physical Stresses

Tumors disrupt the mechanical homeostasis of normal tissues and induce physical stresses in the TME (71), which include solid and fluid stresses (e.g., compressive, tensile, and shear). Tumor growth fueled by cell infiltration, proliferation, and ECM deposition may displace existing structures within the TME and exert compressive stress on the surrounding tissue (72). It can also compromise the architecture of blood and lymphatic vessels, leading to an elevated interstitial fluid pressure over the surrounding tissue (73). The pressure gradient drives fluid flow and shear stresses on the tumor cells. These stresses can modulate tumor cell behavior, including angiogenesis, metabolic reprogramming, and metastasis (74, 75). Physical stresses of the TME have been recapitulated in microfabricated models using pneumatic actuators (76), vacuums (77), and gravity-driven mechanisms (78). Perfusion of microfabricated models, where cell culture medium continuously flows through the culture chamber/channels, exposes cultured cells to shear stress, mimicking flow-induced shear stress typical of blood vessels (79). Perfusion culture requires additional components, including tubing, connectors, reservoirs, and pumps, which may give rise to complications, especially during long-term culture, including leakage, bubbles, and drying of media. Therefore, optimization of the perfusion culture condition is needed.

## 4. MICROFABRICATED TISSUE- AND ORGAN-SPECIFIC TME MODELS

Cancers originating from different cell and tissue types demonstrate vast differences in response to even the same oncogenic driver mutations (80). Different organs also have unique combinations of stromal cells (81, 82), ECM proteins (83), and resident immune cells (84, 85). Therefore, developing organ-specific microfabricated tumor models is imperative to understand their distinct biology and evaluate unique therapeutic responses for next-generation cancer medicine. Here, we highlight some of the most recently developed organ-specific primary tumor models, with a focus on four organ types of origin—lung, liver, brain, and bone—which all have low 5-year relative survival rates in patients (86).

### 4.1. Lung Tumor

#### 4.1.1. Lung.

**Figure 2:**
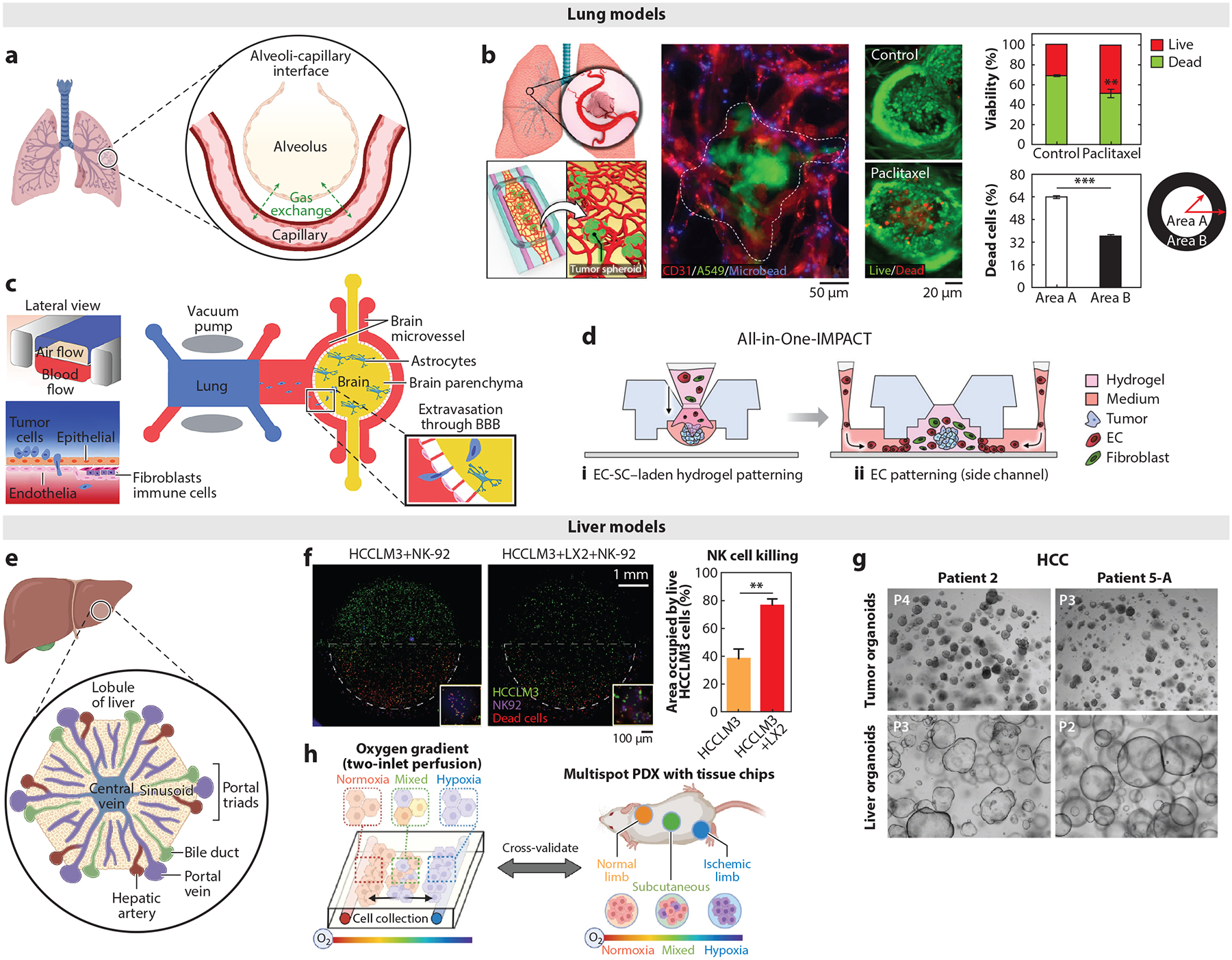
Relevant organ structure and microfabricated lung- and liver-specific TME models. (*a*–*d*) Lung models. (*a*) Lung anatomy and physiology. Panel adapted from images created in BioRender; Oh, J. 2025. https://BioRender.com/b26f027. (*b*) Human vascularized solid lung tumor-on-a-chip for high-content drug screening. Tumor spheroids made from lung cancer cells (*green*) and endothelial cells (*red*) are cultured within a hydrogel containing endothelial cells and fibroblasts, which vascularize the tumor spheroids. Microbeads (*blue*) are introduced to the established vasculature to confirm its perfusability. Paclitaxel is delivered through the established vasculature, and its tumor-killing effect is demonstrated. Panel adapted with permission from Reference 87; copyright 2019 American Chemical Society. (*c*) Schematic of the multiorgan microfluidic chip for modeling brain metastasis of lung cancer cells. Panel adapted from Reference 88 (CC BY-NC-ND 4.0). (*d*) Concept and technical advantage of All-in-One-IMPACT platform for establishing vascularized tumor spheroid models. Panel adapted with permission from Reference 89; copyright 2022 Wiley Periodicals LLC. (*e*–*h*) Liver models. (*e*) Liver anatomy and physiology. Panel adapted from images created in BioRender; Oh, J. 2025. https://BioRender.com/t49q016. (*f*) Biomimetic platform for recapitulating liver cancer (HCC-on-a-chip), and evaluation of NK-92 cell cytotoxicity against HCC cell HCCLM3 with or without human hepatic stellate cells. Panel adapted from Reference 90 (CC BY-NC-ND 4.0). (*g*) Establishing HCC organoids from needle biopsies of HCC patients. Panel adapted from Reference 91 (CC BY-NC-ND 4.0). (*h*) Schematic of dual oxygen gradient chip for elucidating oxygen gradient–induced intratumoral and interpatient heterogeneity in HCC. Panel adapted from Reference 92 (CC BY 4.0). Abbreviations: BBB, blood–brain barrier; EC, endothelial cell; HCC, hepatocellular carcinoma; NK, natural killer; PDX, patient-derived xenograft; SC, stromal cell; TME, tumor microenvironment.

The primary function of the lung is to facilitate the exchange of oxygen and carbon dioxide between air and the blood in pulmonary capillaries (93) (Figure 2*a*). The lung is composed of a complex network of branching airways that progressively divide into smaller tubes, ultimately leading to thin, grape-like clusters of small air sacs called alveoli. Compared to their size (diameter 50–100 μm), alveoli have a very thin membrane (0.5 μm) and are surrounded by a dense capillary network, which allows for the efficient diffusion of oxygen into the bloodstream and the removal of carbon dioxide (94). The cellular composition of the alveoli–capillary interface includes epithelial cells, endothelial cells, fibroblasts, and immune cells (predominantly macrophages that respond to foreign substances) (94). Alveoli are subjected to multiple mechanical cues during gas exchange, including cyclic stretching and blood circulation. The cyclic stretching results in approximately 5–12% strains at 0.2 Hz in physiological conditions (95).

Lung cancers account for 13% of all cancers and 20% of all cancer deaths, making it the leading cause of cancer-related death worldwide (96). Lung cancers are broadly classified into small cell lung cancer (SCLC) and non–small cell lung cancer (NSCLC), which is further classified into specific subtypes (97). Genetic abnormalities observed in lung cancer are specific to the subtype, with *EGFR* and *KRAS* mutations and increased CD44 and EGFR protein expression commonly observed in NSCLCs versus *MYC* amplification, *TP53* and *RB1* mutations, and increased BCL2 and c-KIT protein expression observed in SCLCs (98, 99). NSCLCs are the most prevalent type of lung cancer, accounting for 85% of all lung cancers. Consequently, significant endeavors are underway to deepen the comprehension of NSCLC development and target therapy resistance mechanisms in this disease. Notably, the development of lung cancer can significantly disrupt the anatomy and physiology of the affected lung. NSCLCs commonly exhibit excessive growth of fibrous tissue around the tumor, which adds ECMs (e.g., laminin and collagen) that are stiffer than the normal lung stroma to the site (94, 100).

#### 4.1.2. Models of lung TME.

Paek et al. (87) developed an in vitro platform that enables the integration of a perfusable, self-assembled microvasculature with 3D lung tumor spheroids (i.e., a vascularized solid tumor-on-a-chip) (Figure 2*b*). In this study, multicellular tumor spheroids were formed using human lung adenocarcinoma cells (A549) and endothelial cells [human umbilical vein endothelial cells (HUVECs)] and embedded in an ECM hydrogel scaffold that contained endothelial cells and lung fibroblasts (Figure 2*b*). Spheroids maintained structural integrity while growing larger during culture and were fully integrated with the surrounding microvessels. Clinical concentrations of paclitaxel were administered to the culture through the perfusable vasculature to simulate intravascular drug delivery to lung tumors and to assess chemotherapy-induced vascular toxicities. Their model demonstrated that paclitaxel can induce endothelial oxidative stress and inflammation, which are key potential mechanisms of paclitaxel-induced vascular toxicities (101).

Liu et al. (88) developed a multiorgan microfluidic chip consisting of two organ units, an upstream “breathing” lung and a brain at the downstream, characterized by a functional blood–brain barrier (BBB), to understand the mechanisms underlying brain metastasis of NSCLC (Figure 2*c*). The lung tumor model contained a human lung cancer cell line (PC9), as well as human bronchial epithelial cells (16HBE), pulmonary microvascular endothelial cells, lung fibroblasts (HFL1), and mononuclear cells (THP-1), whereas the brain tumor model utilized human astrocytes (HA-1800) and brain microvascular endothelial cells for the cerebral BBB structure. All the parenchymal and stromal cells in the lung model originated from the lung to capture the organ-specific cellular characteristics. Furthermore, multiple mechanical signals, including stretching that mimics breathing and continuous media flow that mimics blood circulation, were applied in the multiorgan system. PC9 cells with differing metastatic capacities were observed to migrate from the lung model to the brain parenchyma after extravasating through the BBB. Using this model, they demonstrated the important role of aldo-keto reductase family 1 B10 (AKR1B10) in forming brain metastasis by lung cancer cells.

Liquid patterning has been integrated into microfluidic culture platforms to enhance the throughput of microfabricated tumor models. Kim et al. (89) developed an injection-molded, all-in-one culture platform (i.e., All-in-One-IMPACT) that enables the formation of vascularized tumor spheroids through fluid patterning with patient-derived cancer cells and various cancer cell lines, including lung cancer cells (A549). Compared with the multiple manual handling and transfer steps used in the traditional methods of establishing vascularized tumor spheroids, the All-in-One-IMPACT platform utilized a microfluidic design that streamlined the workflow to just two steps (Figure 2*d*), which minimized handling errors and enhanced reproducibility and throughput. This platform successfully established vascularizing tumor spheroid models of lung cancer (using A549), liver cancer (using HEPG2), and gastric cancer (using patient-derived cells). They observed distinct organ-specific heterogeneity of vessel areas in the vascular network established in the TME of different cancer models.

### 4.2. Liver Tumor

#### 4.2.1. Liver.

The liver performs diverse functions that are essential for the proper function of the human body, including macronutrient (e.g., glucose, lipid, protein) metabolism, blood volume regulation, immune system support, and detoxification (102). These functions are mainly carried out by hepatocytes, which constitute 60% of the liver cell populations (103). Other cell types include endothelial cells, hepatic stellate cells (HSCs), biliary epithelial cells, Kupffer cells (liver-resident macrophages), and other immune cells. The basic functional unit of the liver is referred to as the lobule, which is represented as chords of hepatocytes organized in a hexagonal shape around the central vein (102) (Figure 2*e*). This spatial organization establishes gradients of oxygen, nutrients, and other soluble factors from the edge to the center of the lobule. The liver is highly vascularized in a unique pattern, where afferent (supplying) and efferent (draining) blood vessels interdigitate uniformly (104).

The development of liver cancer can significantly disrupt the anatomy and the physiology of the affected liver (105). Liver cancer is the sixth most common cancer and the fourth leading cause of cancer-related death globally (106). Hepatocellular carcinoma (HCC), which develops from hepatocytes, accounts for 90% of all primary liver cancer incidents (106). Surgical resection and liver transplantation remain the cornerstone curative approaches for HCC cases (107). Common cancer drivers in HCC include mutations of *TERT*, *TP53*, and *CTNNB1* (106). HCC occurs frequently in people with chronic liver diseases, such as cirrhosis (i.e., severe scarring of the liver) caused by hepatitis B or C infection. Chronically activated HSCs play a crucial role in the HCC TME; they assume a myofibroblast phenotype, deposit and organize ECM proteins in the injured liver, and drive HCC tumorigenesis and progression (108). Other cellular components of the HCC TME include stromal hepatocytes, CAFs, MDSCs, and TAMs (102, 108). The noncellular components include cytokines (e.g., IL-6, IL-22), growth factors (VEGF, TGF-β, PDGF), matrix metalloproteinases, and proteoglycans (108).

#### 4.2.2. Models of liver TME.

The microfluidic HCC model developed by Shen et al. (90) highlighted the cross talk between activated HSCs and HCC cells in the HCC TME (Figure 2*f*). The polydimethylsiloxane (PDMS)-based microfluidic device utilized in the HCC model comprised three chambers separated by trapezoidal barriers and a central chamber surrounded by two lateral channels. The HCC cells (HCCLM3) and HSCs (LX2) were mixed at 1:1 ratio in Matrigel and embedded in the central chamber. Compared with the LX2 monoculture, LX2 cells cocultured with HCC cells upregulated the expression of multiple genes associated with HSC activation, including *ATA2*, *COL1A1*, and *COL1A4*, as well as signaling pathways related to cell adhesion and ECM remodeling. Furthermore, coculturing HSCs with HCCs in the model promoted endothelial invasion into the tumor bulk, enhanced sorafenib resistance, and decreased NK cell (NK-92) infiltration and NK-mediated tumor killing. RNA sequencing and secretome analysis on the established HCC model identified LCN-2 as a key factor in HCC development, which was further validated with clinical samples and databases.

Recently, patient-derived samples have been incorporated into the HCC models to better reflect the conditions of the originating patients. Nuciforo et al. (91) developed HCC organoids and nontumor liver tissue organoids from needle biopsies obtained from HCC patients to serve as a tool for developing patient-specific therapies (Figure 2*g*). The histological characteristics of the originating tumors, including the growth patterns and differentiation grades, were preserved in the generated HCC organoids. Furthermore, whole-exome sequencing of the HCC organoids, along with their paired tumor and nontumor biopsies from patients, confirmed that the HCC organoids retained the somatic genetic alterations of the originating tumor. The diverse oxygen gradients found within the HCC TME, established by a dual blood supply from the hepatic artery (high oxygen concentration) and portal vein (low oxygen concentration), contribute to the heterogeneous nature of HCC. Baek et al. (92) developed a dual gradient chip that facilitates the simultaneous perfusion of normoxic and hypoxic media (oxygen gradient) and media with and without drugs (drug gradient) through separate inlets (Figure 2*h*). HCC tissue pieces from 12 patients were mixed with gelatin hydrogel and embedded in the chip for oxygen gradient culture. Strikingly, each HCC tissue retained the inherent tissue characteristics related to oxygen preference, where HCC from well-oxygenated tissue demonstrated higher viability on the normoxic side of the chip, while HCC from hypoxic tissue demonstrated preserved viability under hypoxic conditions.

### 4.3. Brain Tumor

#### 4.3.1. Brain.

**Figure 3:**
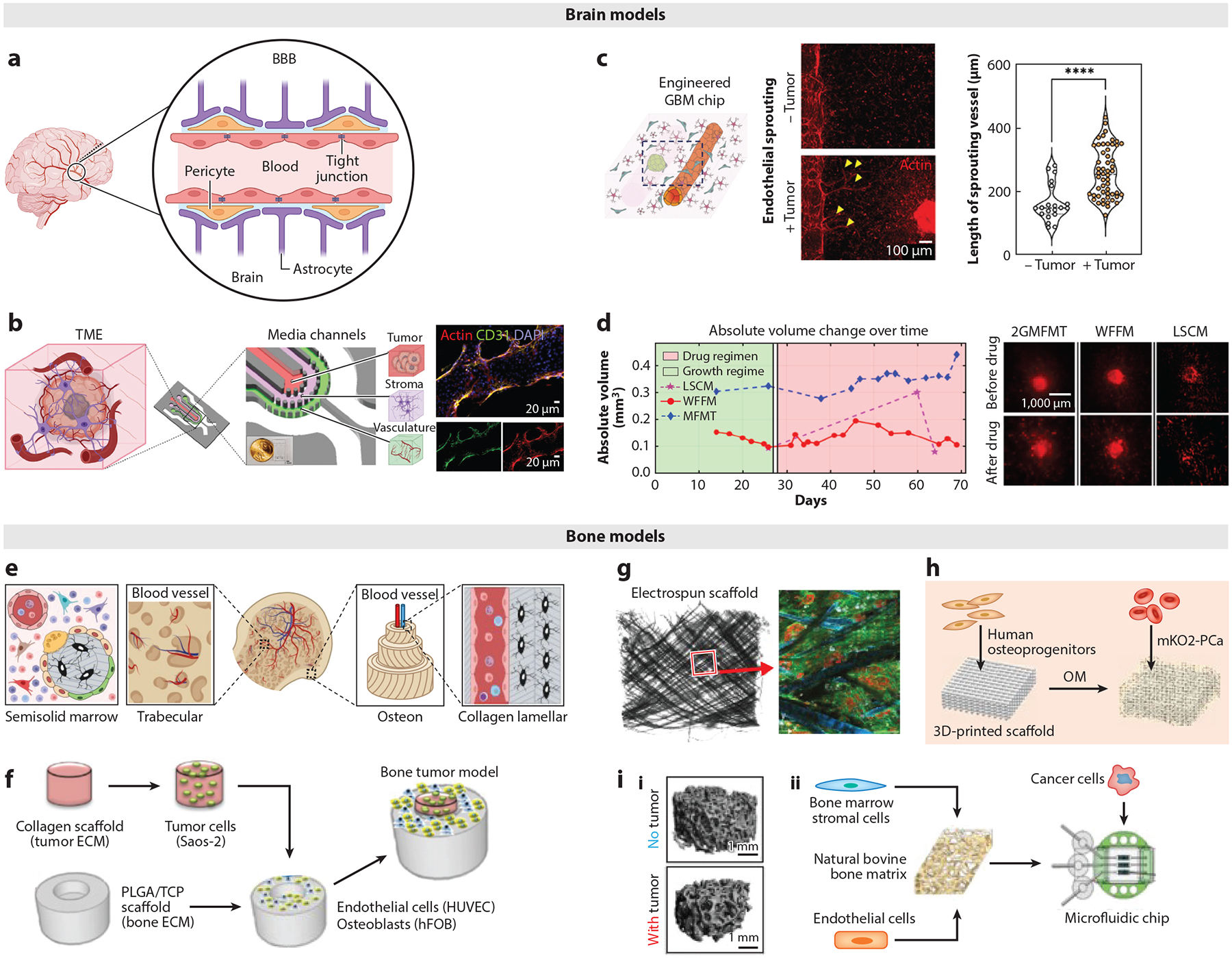
Relevant organ structure and microfabricated brain- and bone-specific TME models. (*a*–*d*) Brain models. (*a*) Brain anatomy and physiology. Panel adapted from images created in BioRender; Oh, J. 2025. https://BioRender.com/j84i307. (*b*) Microdevice for recapitulating the PVN within the GBM. The microdevice has three compartments: tumor, stroma, and vascular (fluorescence image). Panel adapted from Reference 109 (CC BY 4.0). (*c*) Model of 3D human GBM integrated with triculture BBB (human brain endothelial cells, human astrocytes, and human brain vascular pericytes), and schematic of the engineered GBM-BBB model, where GBM-induced angiogenesis is observed. Panel adapted from Reference 110 (CC BY 4.0). (*d*) 3D-bioprinted GBM model integrated with a noninvasive, fast, deep-tissue imaging system. The captured images are 3D reconstructed for longitudinal volumetric assessment before and after drug treatment. Panel adapted from Reference 111 (CC BY-NC 4.0). (*e*–*h*) Bone models. (*e*) Hierarchical structure of bone tissue. Trabecular bone has a porous cavity structure. Compact bone is composed of dense collagen lamellae. Panel adapted from images created in BioRender; Lee, J. 2025. https://BioRender.com/w51f254. (*f*) Two-compartment scaffold mimics healthy bone and tumor tissue, which recapitulates the tumor progression near bone tissue. Panel adapted from Reference 112 (CC BY 4.0). (*g*) 3D electrospun and (*h*) 3D-printed scaffolds reproduced bone tissue complexity; subsequently added human prostate tumor cells reproduced bone tumor progression. Panel *g* adapted from Reference 113; copyright 2019 Elsevier Ltd. Panel *h* adapted from Reference 114 (CC BY-NC 4.0). (*i*) Natural bone-based models: (*i*) Bovine decellularized trabecular bone demonstrating osteolysis by human osteosarcoma. Panel adapted from Reference 115; copyright 2017 Mary Ann Liebert, Inc. (*ii*) Microfluidic chip including a 3D trabecular bone recapitulating bone tumor development. Panel adapted from Reference 116 (CC BY 4.0). Abbreviations: 2GMFMT, second-generation mesoscopic fluorescence molecular tomography; BBB, blood–brain barrier; ECM, extracellular matrix; GBM, glioblastoma multiforme; hFOB, human fetal osteoblast; HUVEC, human umbilical vein endothelial cell; LSCM, laser scanning confocal microscopy; MFMT, mesoscopic fluorescence molecular tomography; OM, osteogenic medium; PLGA, poly(lactic-co-glycolic) acid; PVN, perivascular niche; TCP, tricalcium phosphate; TME, tumor microenvironment; WFFM, wide-field fluorescence microscopy.

The human brain is one of the most complex organs in the human body and serves as the control center of the nervous system (117). The brain consists of neurons and non-neuronal glial cells, including astrocytes, oligodendrocyte progenitor cells, oligodendrocytes, microglia, and cells of the brain vasculature (118). Neurons are information-processing cells, whereas glial cells perform specific functions that support overall brain development and activity (119). Notably, the proper function of the brain relies on the selective transport of nutrients, molecules, and cells through the BBB (120) (Figure 3*a*). The BBB is composed of specialized endothelial cells with extensive tight junctions that form the capillaries, along with pericytes and astrocytes. The BBB protects the brain parenchyma from blood-borne agents and inflammatory molecules; it also forms a significant obstacle to the delivery of drugs and other therapeutic compounds into the brain. The ECM of the brain is significantly different from other organs in the human body (121). It mainly consists of proteoglycans, glycoproteins, and glycosaminoglycans, which include heparan sulfate proteoglycans and hyaluronic acid.

Glioblastoma multiforme (GBM) is the most aggressive and malignant form of brain cancer, with a 5-year survival rate of 7.2%. The majority of the immune cells found within the brain TME are macrophages of ontogenetically distinct populations, including brain-resident microglia and bone marrow–derived macrophages. Neuronal activity has been observed to promote high-grade glioma proliferation through upregulation of neuroligin-3 (NLGN3) (122). The development of brain tumor modifies the BBB, which is referred to as the blood–tumor barrier (BTB) and features a loss of tight junctions in the endothelium (123). Furthermore, the BTB also has disrupted adherens junctions, overexpressed TNF receptors 1 and 2, and downregulated expression of many uptake and efflux transporters (124). Additional brain-specific TME features, including changes in the brain matrix and neuron function, are highlighted by Quail et al. (125).

#### 4.3.2. Models of brain TME.

Numerous brain tumor models have been developed to mimic the TME of GBM, particularly in relation to glioblastoma stem cells (GSCs) due to their inherent resistance to conventional therapies (126). These models provide valuable insights into GSC behavior, aiding in the development of innovative therapeutic strategies. Cornelison et al. (127) introduced a 3D in vitro model of a human GBM TME by incorporating patient-derived GBM stem cells, human primary cortical astrocytes, human SV40-immortalized microglia, and interstitial flow. To increase the physiological relevance of the recapitulated GBM TME, they analyzed the cellular composition of GBM patient resection samples. They determined that a cell ratio of 6:1:1 for glioma:astrocyte:microglia reflected the cellular composition of the general GBM patients. The cell mixture was embedded within a hyaluronan-based hydrogel, and interstitial fluid flow was applied. Experimenting with seven patient-derived GSC lines, they confirmed patient heterogeneity in GSC invasion and proliferation. They also demonstrated the impact of glioma–glial cell interactions and interstitial fluid flow on glioma cell expression of the stemness marker CD71. Adjei-Sowah et al. (109) used an organotypic microfluidic tumor model to examine glioma stem cells in the perivascular niche (PVN) at single-cell resolution (Figure 3*b*). Their device featured three concentric cell culture regions representing the tumor (GB3 cells in Matrigel), stroma (human astrocytes in Matrigel), and vasculature (HUVECs in fibrin), surrounded by media channels. They showed that astrocytes in the PVN created migratory tracts and secreted ligands, inducing chemotaxis and contributing to high GB3 invasion.

The BBB is altered with the development of GBM, and its modified function contributes to poor therapeutic efficacy (123). The human BBB model developed by Seo et al. (110) utilized human brain vascular pericytes and human astrocytes to represent the brain tissue, and human brain microvascular endothelial cells were seeded in a hollow channel generated by microneedles (Figure 3*c*). All cell sources in this model originated from the human brain, which addresses the drawbacks of the other existing BBB models that utilize nonbrain specific cells (e.g., HUVECs and human lung fibroblasts). Human GBM spheroids, created using two types of human GBM cell lines (T98G and U87MG), were introduced to the BBB model to study the interaction of the BBB and human GBM. With the introduction of the GBM spheroids, they observed significant endothelial sprouting of the brain endothelial cells toward the spheroids. Furthermore, the blood vessels were significantly enlarged, were more permeable, and expressed ICAM1 in the presence of GBM spheroids. Morphological and behavioral changes were observed in T98G cells in the presence of the BBB due to increased production of cytokines associated with tumor invasion and growth (e.g., IL-2, −5, −6, −7, −10, −13, −15, and −21). After observing the reduced efficacy of anticancer drugs vincristine and doxorubicin (DOX) due to the BBB hindrance, they confirmed enhanced delivery and efficacy of DOX when combined with BBB-opening agents, mannitol, and gintonin.

Tang et al. (128) developed a customized digital light processing–based 3D-printing system with GSCs, human astrocytes, neural progenitor cells (NPCs) (such as hNP1s), and macrophages (THP-1 derived, human induced pluripotent stem cell–derived, or primary human macrophages) embedded in photo-cross-linkable gelatin methacylate (GelMA) and glycidyl methacrylate-hyaluronic acid (GMHA) hydrogels. The developed 3D-printing system utilized a digital micromirror device chip to control the light projection to a specified area. They mixed and printed M2 macrophages with GSCs at the center with astrocytes and NPCs surrounding the central tumor core. Compared with the GBM model without macrophages, the GSCs in the GBM model with macrophages upregulated activation of hypoxia and glycolytic metabolism signatures and proinvasive transcriptional profiles. Furthermore, compared with the traditional suspension cultured macrophages, the GBM model cultured macrophages showed regulation of defense response genes, including *CH14*, *PAL2G7*, and *ALOX5*, and M2 macrophage-related markers, including *CD163* and *IL-10*. Ozturk et al. (111) developed a 3D imaging modality [i.e., second-generation mesoscopic fluorescence molecular tomography (2GMFMT)] that enables the noninvasive assessment of longitudinal fluorescent signals over their whole GBM model with perfusable vascular channels (Figure 3*d*). A patient-derived GBM spheroid was placed between two vascular channels lined with HUVECs and cultured under dynamic perfusion for up to 70 days. The developed imaging system, 2GMFMT, supported a higher temporal resolution compared with the traditional imaging modalities and enabled long-term monitoring of tumor invasion.

### 4.4. Bone Tumor

#### 4.4.1. Bone.

Anatomically, bone can be classified into inner trabecular and outer compact bone (129, 130). Trabecular bone has a spongy-like porous architecture, providing structural support and flexibility by dampening the mechanical stress. It contains richly vascularized semisolid marrow that reserves hematopoietic stem cells and stromal cells that support hematopoiesis (131, 132). Compact bone provides greater strength and rigidity than trabecular bone. Its fundamental functional and structural unit is an osteon, consisting of concentric lamellae layers that surround a hollow passageway for vascular canals without gaps (133). From an ECM perspective, bone is a composite organ consisting of organic (mainly type I collagen) and inorganic (mainly hydroxyapatite) biomaterials. Collagen fibrils, assembled from type I collagen molecules, have hydroxyapatite nanocrystals deposited along them (134). The well-aligned collagen fibers serve as templates for hydroxyapatite nucleation and propagation. The structural hierarchy of collagen fibers and hydroxyapatite from nano- to macroscale provides the unique mechanical properties and flexibility of bone (135, 136) (Figure 3*e*). On a cellular level, bone-forming osteoblasts and bone-resorbing osteoclasts continually remodel the bone surface to maintain mechanical structure and mineral homeostasis. Subsurface residing osteocytes comprising more than 90% of total bone cells regulate the bone surface metabolism via paracrine signaling in response to mechanical stimulation (137).

Primary bone tumors are less common and less metastatic than primary lung, liver, and brain tumors. Osteosarcoma, arising from osteoblasts in osteoid tissues, is the most prevalent malignant bone tumor in children and the third most common cancer among adolescents. It predominantly affects the long bones near the shoulder and knee, but it can occur in any bone, especially in older adults (138). Osteosarcoma remains a significant concern as it can invade nearby skeletal tissue, causing osteolysis and making the bone more susceptible to fractures. Despite advancements in treatment, up to 40% of patients with primary bone tumor experience metastasis, often to the lungs (139). Secondary bone tumors are further discussed in Section 5.

#### 4.4.2. Models of bone TME.

Numerous biomaterials (synthetic, natural, hybridized) and processing techniques have been developed for 3D microfabricated bone tissue models to recapitulate the extracellular and structural complexity of bone tissue with relevant cell types (140). These bone tissue models have been applied to study bone TME and anticancer drug responses, which can be classified into four different approaches. First, microfabricated 3D porous scaffolds have been developed to replicate a spongy-like trabecular bone structure (Figure 3*f*). For example, a PLGA/beta-tricalcium phosphate (β-TCP) scaffold was prepared using sodium chloride particles as porogen (templates to create pores). The scaffold supported the proliferation and differentiation of human osteogenic progenitors to form a bone tissue mimic. The tumor component was separately established in a collagen scaffold prepared by a freeze-drying (or lyophilization) method and subsequently seeded with tumor cells. By combining the PLGA/β-TCP and collagen scaffolds, this study recapitulated the interaction between bone and tumor cells, as well as the migration tendency of tumor cells between the bone and tumor sites (112). This two-compartment scaffold is a promising strategy to reconstitute bone and marrow tissue complexity while studying the effects of bone TME in directing tumor progression and drug resistance.

Second, electrospun 3D scaffolds have been used to replicate the well-organized bone collagenous ECM and better induce the osteogenic differentiation of bone marrow stromal cells (BMSCs) (Figure 3*g*). For example, a polycaprolactone-based electrospun mesh scaffold supported osteoblasts to form mineralized bone tissue analogs. By inoculating cancer cells into these scaffolds, the bone TME was simulated. Advanced microscopy, time-course imaging, and imaging analysis algorithms enabled detailed investigation of tumor and stromal interactions, providing insights into how cancer cells respond to the combination of radiation treatment and chemotherapy. This bone TME model also demonstrated stromal-dependent resistance of tumor cells to these cancer therapies (113).

Third, 3D-printing techniques have been utilized to create 3D bone tissue models with predesigned multiscale 3D architecture (Figure 3*h*). For instance, Bock et al. (114) fabricated highly precise and reproducible 3D porous scaffolds using melt electrowriting and 3D-printing methods. The scaffolds combined with calcium phosphate promoted the proliferation and differentiation of human osteoblasts, forming a mineralized bone tissue microenvironment. When human cancer cells were cocultured, this study demonstrated the ability to substantiate the crucial factors of tumor growth within the mineralized bone TME (114).

Lastly, the natural bone matrix itself has been used to create bone TME because, despite significant advances in biomaterials and fabrication methods, replicating the intrinsic hierarchical composite structure of the bone ECM remains challenging. For example, Villasante et al. (115) reconstituted bone tissue complexity by culturing human osteoblasts on a decellularized bovine trabecular bone matrix. Subsequently introduced human CD14^+^ cells supplemented with RANKL differentiated them into osteoclasts. By incorporating human osteoclasts and osteoblasts into the natural bone matrix, this model recapitulated the bone remodeling and demonstrated the effect of zoledronic acid (a bisphosphonate-based mineral binding drug) that inhibits the bone resorption mediated by osteoclasts in the treatment of Ewing’s sarcoma (115) (Figure 3*i*, **subpanel *i***). A decellularized trabecular bone matrix was also integrated with a microfluidic chamber to replicate the perivascular niche via culturing BMSCs and endothelial cells under medium perfusion. The interstitial flow significantly promoted angiogenesis, resembling the perivascular niche in the bone marrow. Subsequently introduced cancer cells simulated bone TME, allowing for monitoring of the colonization of cancer cells in the context of bone and marrow and their responses to cancer drugs under physiologically relevant culture conditions (116) (Figure 3*i*, **subpanel *ii***).

Clinical bone explants are very limited to obtain; thus, it is impractical to use them for bone TME studies. Successfully microfabricated bone TMEs are expected to greatly facilitate the investigation of bone tumor progression and the development of effective treatments. While these bone TME models have demonstrated potential, they are limited in their ability to faithfully reconstitute essential bone tissue complexity and recapitulate multicellular processes. Currently, there is no widely accepted bone TME model. The development of relevant, practical, and enabling bone TME models remains an active research area.

## 5. MICROFABRICATED METASTASIS TME MODELS

**Figure 4:**
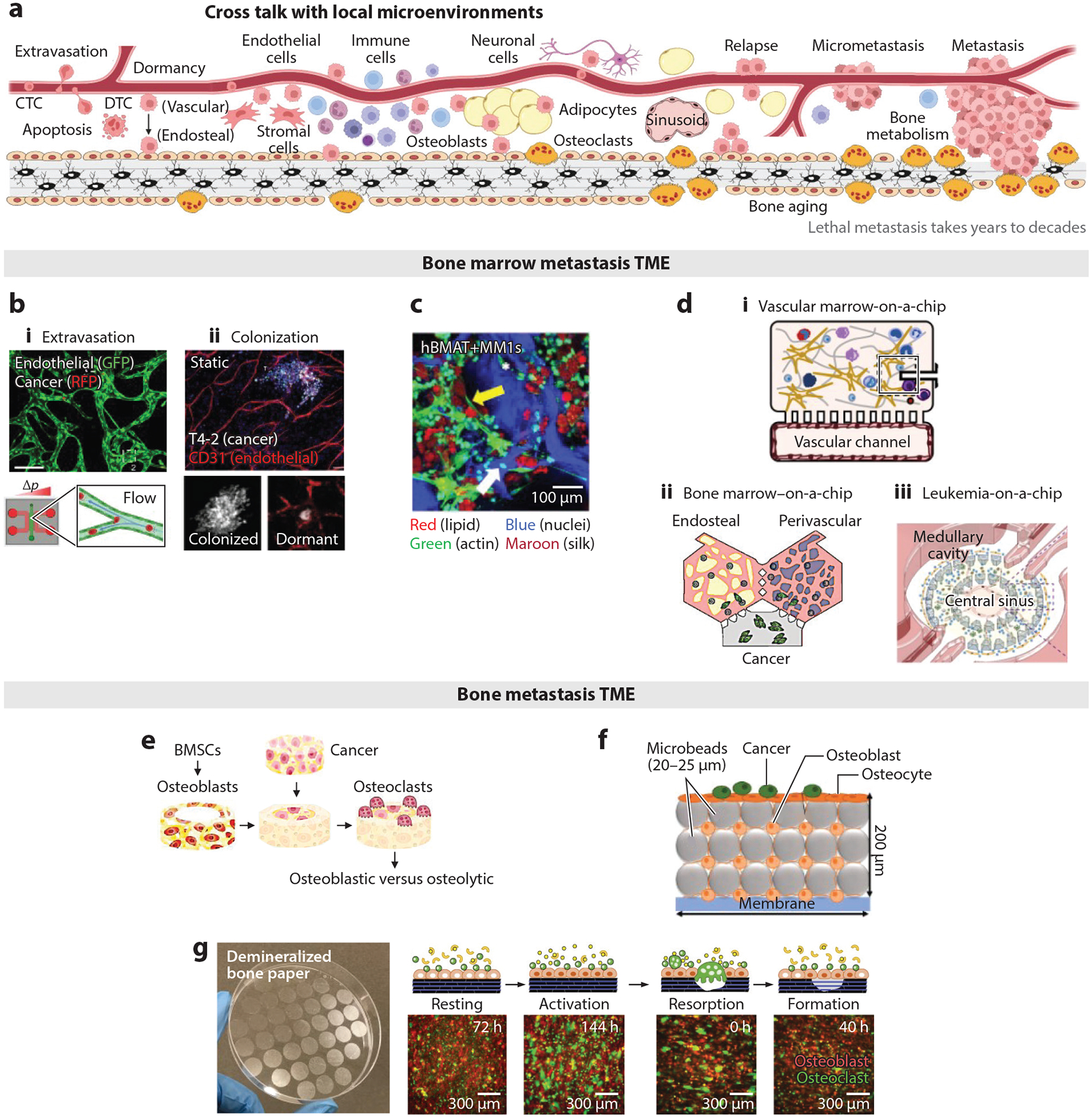
Microfabricated bone and marrow TME models. (*a*) Schematic of tumor cell dissemination, dormancy, and relapse while interacting with local tissue cellular, extracellular, and molecular microenvironments. Panel adapted from images created in BioRender; Lee, J. 2025. https://BioRender.com/k17w541. (*b*–*e*) Bone marrow TME models. (*b*) Bone marrow vascular TME models to recapitulate early-stage metastasis: (*i*) A perfusable vascular chip reproduces the extravasation of CTCs while allowing time-course fluorescent monitoring. Panel adapted from Reference 141 (CC BY 4.0). (*ii*) A perivascular niche model, with and without BMSCs, reproduced the dormancy of DTCs and the role of endothelial cells. Panel adapted from Reference 142; copyright 2013 Macmillan Publishers Ltd. (*c*) Scaffold-based bone marrow stromal TME to study the role of adipocytes: Silk-based scaffolds supported the coculture of bone marrow adipocytes and MM cells, demonstrating a decrease in the size of fat droplets in contact with cancer cells. Panel adapted from Reference 143 (CC BY 4.0). (*d*) Chip-based bone marrow TME models to recapitulate vascular and endosteal niche complexities: (*i*) A vascular marrow-on-a-chip model consisting of marrow and vascular channels replicated clinically relevant hematopoietic toxicities. Panel adapted from Reference 144; copyright 2020 Springer Nature Limited. (*ii*) Compartmentalized endosteal and vascular niches connected via perfusable vascular networks recapitulated the vascularized bone marrow metastasis TME. Panel adapted from Reference 145; copyright 2021 Elsevier Ltd. (*iii*) Microstructures in a chip created bone marrow–mimicking central sinusoid, medullary cavity, and endosteal regions, which supported the in vivo–relevant replication of leukemia cell growth and chemoresistance. Panel adapted from Reference 146 (CC BY-NC 4.0). (*e–g*) Bone TME models. (*e*) Triculture model consisting of BMSC-derived osteoblasts, THP-1-derived osteoclasts, and breast cancer cells (MDA-231) recapitulated bone metastatic TME and demonstrated cancer cell–dependent osteolytic and osteoblastic lesions. Panel adapted from Reference 147; copyright 2023 Elsevier Ltd. (*f*) A microfluidic perfusion chamber filled with microbeads and osteocytes simulated in vivo–like ECM complexity and mechanical milieu. Subsequently introduced osteoblasts and prostate cancer cells recapitulated the bone metastatic TME. Panel adapted from Reference 148 (CC BY 4.0). (*g*) An osteoid-mimicking demineralized bone paper preserves the intrinsic collagen structure of the bone matrix while retaining durability and semitransparency. Recapitulation of a bone remodeling cycle via coculture of reporter murine osteoblasts and bone marrow monocytes on demineralized bone paper under biochemical stimulation is demonstrated, with longitudinal fluorescent monitoring. Panel adapted from Reference 149 (CC BY-NC 4.0) and Reference 150 (CC BY 4.0). Abbreviations: BMSC, bone marrow stromal cell; CTC, circulating tumor cell; DTC, disseminated tumor cell; ECM, extracellular matrix; GFP, green fluorescent protein; hBMAT, human bone marrow adipose tissue; MM, multiple myeloma; RFP, red fluorescent protein, TME, tumor microenvironment.

Metastasis is the leading cause of death from nearly all cancers, but the underlying mechanisms remain largely uncertain due to the complex and lengthy nature of the process. This cascade includes the circulation and dissemination of tumor cells, survival in distal tissues and organs, entering and awakening from dormancy, and eventual lethal metastasis. Many factors can affect these processes, including advanced aging, cancer therapies, lifestyle factors (diet and exercise), and environmental influences. However, detailed investigation of metastatic TMEs is difficult in animal models and clinical studies. Microfabricated organ-specific TMEs hold great potential to simulate essential tissue complexity and multicellular processes while allowing longitudinal monitoring of tumor cell behaviors and their response to therapeutic interventions. In this section, we focus on bone metastasis TME, which is particularly difficult to monitor due to the limited anatomical accessibility of the inner space of bone cavities. We divide the bone TME into the vascularized semisolid marrow and the bone surface undergoing repeated remodeling (Figure 4*a*).

### 5.1. Models of Bone Marrow Metastasis

CTC extravasation to the bone marrow vasculature holds the potential for therapeutic intervention in bone metastasis. Microfabricated fluidic chips that mimic perfusable vascular networks have allowed for a detailed investigation of this process. Endothelial cells cultured in a gel-containing chip with growth factors differentiate into perfusable vascular conduits under hydrostatic pressure. Introducing fluorescent-labeled tumor cells into the perfused medium allows monitoring of dynamic extravasation processes through time-course 3D confocal imaging. This approach also facilitates a detailed analysis of the local cellular and extracellular components involved in extravasation. For example, the vascular chip model substantiated glycocalyx-mediated extravasation of CTCs, identified as a significant driver of cancer progression. This study also unveiled the essential role of hyaluronic acid, shed by CTCs, in mediating adhesion to the endothelium through the glycoprotein CD44 (141) (Figure 4*b*, **subpanel *i***). Successfully extravasated and survived DTCs in the bone marrow can remain dormant for varying periods before resuming proliferation. While the existence of dormant DTCs is well established, the factors that induce and maintain DTC dormancy remain an active research area. An elaborate study using engineered microvascular models demonstrated significant suppression of DTC growth in coculture with endothelial cells and BMSCs compared with BMSC single culture. This study further substantiated the role of endothelial-derived thrombospondin-1 in sustaining DTC quiescence (142) (Figure 4*b*, **subpanel *ii***). Microfabricated bone marrow vascular models are promising in facilitating preclinical investigations of early-stage bone metastasis and the functional and dynamic interaction of DTCs with the vascular niche.

Adipocytes are another major stromal cell type in bone marrow, secreting various adipokines and sequestering lipophilic molecules. A 3D silk scaffold–based adipocyte culture with multiple myeloma cells showed significantly decreased adipocyte droplet size compared with adipocyte single culture (143) (Figure 4*c*). Inverted colloidal crystal hydrogel scaffolds, which closely resemble bone marrow sinusoid architecture and a semisolid biophysical milieu, supported the mature adipocyte differentiation of BMSCs. These 3D bone marrow adipocyte culture models demonstrated the sequestration of doxorubicin, a common hydrophobic chemotherapeutic drug, into adipocyte fat droplets. This sequestration, monitored by exploiting doxorubicin’s autofluorescence, significantly reduced its cytotoxicity. The autofluorescence of doxorubicin decayed over a few days, indicating the metabolization of sequestered doxorubicin, which may alter adipocyte function and the local TME (151). Further detailed investigation of adipocytes in conjunction with other stromal cells represents a promising direction to advance our understanding of the bone marrow TME.

The combination of microscale fabrication and chip-based microfluidic technology has become increasingly powerful in reproducing essential tissue complexity with precise control while allowing excellent microscopic access. These capabilities facilitate better recapitulation of in vivo–relevant physiological processes and investigation into the impact of various therapeutic interventions and tumor progression. One example is a vascularized human bone marrow–on-a-chip model, consisting of compartmentalized vascular and marrow channels. Endothelial cells cultured in the vascular chip under medium perfusion form an endothelial lining. Human BMSCs and CD34^+^ hematopoietic stem cells in the marrow channel are cocultured within a gel matrix. This chip-based bone marrow model supports multilineage differentiation of hematopoietic stem cells and recapitulates the effects of radiation and chemotherapy on bone marrow hematopoietic toxicity (144) (Figure 4*d*, **subpanel *i***). By extending a compartmentalization strategy, chip-based bone marrow models have further demonstrated the inclusion of bone components and the ability to recapitulate both solid and liquid TME. For example, Glaser et al. (145) reported compartmentalized endosteal and perivascular niche models connected via a perfusable vascular network. This model demonstrated the functional ability to support CD34^+^ hematopoietic stem cells, the egress of neutrophils, and niche-specific responses to doxorubicin and granulocyte colony-stimulating factor. Genetically labeled human breast cancer cells (MDA-231) were subsequently introduced to mimic bone metastasis and drug responses with high spatiotemporal resolution (145) (Figure 4*d*, **subpanel *ii***). In another study, Ma et al. (146) demonstrated a leukemia-on-a-chip model by designing a microfabricated chip to compartmentalize the central sinusoid, medullary cavity, and endosteal cells with a perfusable chamber that recapitulates bone marrow tissue complexity. Subsequently introduced B cell acute lymphoblastic leukemia (B-ALL) cells emulated the in vivo disease progression and chemotherapeutic responses. Downstream characterization identified perivascular, endosteal, and hematopoietic niche-derived factors that maintain B-ALL survival and quiescence (146) (Figure 4*d*, **subpanel *iii***). Microfabricated chip-based bone marrow TME models are expected to significantly reduce the critical knowledge gap in understanding the dynamic functional cross talk between DTCs and stromal cells, as well as therapeutic responses, with unprecedented experimental control and analytical power.

### 5.2. Models of Bone Metastasis

Bone undergoes constant remodeling via the paired action of bone-forming osteoblasts and bone-resorbing osteoclasts under the spatiotemporal regulation of suppressive and stimulative secreted molecules. Bone metastasis almost always accompanies osteolytic or osteoblastic lesions, indicating a functional link between bone metastasis and bone metabolism. Understanding this relationship is one of the major goals in the development of microfabricated bone TME models. Recently, Gonzalez Diaz et al. (147) reported this possibility using gelatin microribbon scaffolds that support the osteogenic differentiation of BMSCs. An established cylindrical bone model was filled with various cancer cell–seeded gelatin scaffolds and subsequently introduced THP-1 cell-derived osteoclasts to reconstitute the osteoblast–osteoclast–cancer cell complexity. This triculture system allowed functional cross talk between cancer cells, osteoblasts, and osteoclasts, exploiting changes in mineral deposition as a phenotypic marker. Notably, this study demonstrated tumor cell–dependent osteolytic and osteoblastic lesions (147) (Figure 4*e*).

Another critical cell that regulates bone metabolism and possibly DTC behaviors is the mechanosensory osteocyte, which resides beneath the bone surface. Osteocytes, comprising more than 90% of total bone cells, secrete regulatory molecules in response to mechanical cues, collectively influencing bone surface cellular processes via paracrine signaling. Despite their prevalence, in vitro recapitulation of osteocytes remains an active research area due to the intrinsic challenges of replicating the in vivo–relevant ECM milieu and mechanical stimulation (152). Choudhary et al. (148) demonstrated the potential to partially replicate the osteocyte phenotype in vitro by developing a chip-based perfusion culture model. In this model, microbeads packed with human primary osteocytes and later-introduced osteoblasts reproduced bone surface and subsurface cellular and extracellular complexity. Medium perfusion mimicked the mechanical stimulation of osteocytes. Subsequently, human prostate cancer cells seeded on the osteoblast layer mimicked bone metastasis, allowing the investigation of the role of osteocytes (148) (Figure 4*f*).

Nevertheless, existing microfabricated bone models are limited in their ability to recapitulate the bone remodeling cycle. In the resting phase, osteoblasts remain as bone lining cells, secreting high levels of osteoprotegerin (OPG) and low levels of receptor activator of nuclear factor kappa-B ligand (RANKL), which together suppress bone remodeling. Under biochemical or mechanical stimulation, bone lining cells become active osteoblasts and shift their secretion profiles to induce the differentiation of bone marrow monocytes into multinucleated osteoclasts through cell fusion. Once the osteoclasts complete bone resorption, osteoblasts synthesize new bone matrix by first depositing structural collagen matrix, known as osteoid, and subsequently depositing minerals. During this process, osteoblasts gradually revert to their bone lining cell phase while osteoclasts separate into individual cells, completing the cycle of new mineralized collagen formation. Recently, osteoid-inspired demineralized bone paper (DBP) has demonstrated exciting opportunities to faithfully recapitulate the bone remodeling cycle in coculture with primary osteoblasts and bone marrow monocytes under biochemical stimulation (149, 153) (Figure 4*g*). The semitransparent DBP also allows for longitudinal fluorescent monitoring of these cellular processes (150). Establishing a DBP-based bone model represents a significant opportunity to recapitulate essential bone ECM complexity and the multicellular processes of the bone remodeling cycle, as well as its functional connection with tumor cell biology.

## 6. LIMITATIONS AND FUTURE DIRECTIONS

This review highlights significant advances in the microfabrication of TMEs. Although these models are currently limited to use as substitutes for real human tissues, the potential of advanced microphysiological human tissue models has been formally recognized by the FDA Modernization Act 2.0, which accepts data generated from nonanimal human-relevant models for more advanced and human-relevant models to come to the forefront of preclinical testing. The enactment of the FDA Modernization Act 2.0 has greatly encouraged the development and validation of alternative methods to animal testing, which aligns with the growing need for more humanized models in cancer research. To address this need, we propose the following future directions. First, future work should actively leverage patient-derived cells and induced pluripotent stem cells to create personalized TME models that better reflect the diverse genetic backgrounds of the human population. Second, future research should include extensive validation of established human tissue models to ensure their ability to recapitulate essential tissue-specific processes in a reproducible and analytical manner. While fluorescent microscopy imaging is a powerful tool for real-time visualization and spatial analysis within these platforms, it presents challenges for retrieving soluble factors and cells for subsequent molecular profiling. This limitation poses challenges in conducting comprehensive molecular analyses, such as protein quantification and gene expression profiling, which are critical for understanding the underlying mechanisms of tissue response and drug efficacy. Monitoring ECM changes is a promising strategy. For example, mineral content can be monitored in bone models using label-free bright-field imaging and X-ray transparency. Combining fluorescent and X-ray imaging modalities will be a potent approach for monitoring cellular and extracellular processes in vitro. Addressing these challenges will require the development of new strategies for extracting and analyzing biomolecules from microfabricated models without disrupting the integrity of the models. Ideally, these efforts should generate relevant and functional phenotypic assays, which can be applied to the quantitative assessment of therapeutic efficacy and reproducibility. Third, future work should reduce the translational gap between in vitro models and clinical realities. Despite their reliability, these engineered models often lack the full biological complexity of human tumors, including the interactions with the immune system, vascularization, and cellular heterogeneity. Finally, the human-relevant in vitro model must be able to support large-scale studies involving hundreds of devices that will meaningfully accelerate the drug development process. Microfabricated platforms with high complexity and low scalability are less likely to be adopted beyond academic research settings. Achieving these milestones will require collaborative efforts from interdisciplinary teams, including bioengineers, biologists, clinicians, and data scientists. We believe that microfabricated human TME models hold great potential to significantly reduce critical knowledge gaps, identify new therapeutic targets, and accelerate clinical implementation by supporting high-throughput experiments with high predictive power. Microfabricated human TME models have made significant contributions to basic science and translational medicine, and their future is bright and promising.

## References

[1] Biotechnol. Innov. Org. (BIO), Informa Pharma Intell., Quant. Life Sci. Advis. (QLS). 2021. Clinical development success rates and contributing factors 2011–2020. Rep., BIO, Washington, DC

[2] Han JJ. 2023. FDA Modernization Act 2.0 allows for alternatives to animal testing. Artif. Organs 47:449–50 36762462 10.1111/aor.14503

[3] Virumbrales-Muñoz M, Ayuso JM. 2022. From microfluidics to microphysiological systems: past, present, and future. Organs-on-a-Chip 4:100015

[4] Hagerling C, Casbon A-J, Werb Z. 2015. Balancing the innate immune system in tumor development. Trends Cell Biol. 25:214–20 25444276 10.1016/j.tcb.2014.11.001 PMC4380818

[5] Galli F, Aguilera JV, Palermo B, Markovic SN, Nisticò P, Signore A. 2020. Relevance of immune cell and tumor microenvironment imaging in the new era of immunotherapy. J. Exp. Clin. Cancer Res 39:89 32423420 10.1186/s13046-020-01586-y PMC7236372

[6] Upadhyay S, Sharma N, Gupta KB, Dhiman M. 2018. Role of immune system in tumor progression and carcinogenesis. J. Cell. Biochem 119:5028–42 29327370 10.1002/jcb.26663

[7] Beatty GL, Gladney WL. 2015. Immune escape mechanisms as a guide for cancer immunotherapy. Clin. Cancer Res 21:687–92 25501578 10.1158/1078-0432.CCR-14-1860 PMC4334715

[8] Ness S, Lin S, Gordon JR. 2021. Regulatory dendritic cells, T cell tolerance, and dendritic cell therapy for immunologic disease. Front. Immunol 12:633436 33777019 10.3389/fimmu.2021.633436 PMC7988082

[9] Togashi Y, Shitara K, Nishikawa H. 2019. Regulatory T cells in cancer immunosuppression—implications for anticancer therapy. Nat. Rev. Clin. Oncol 16:356–71 30705439 10.1038/s41571-019-0175-7

[10] Lv M, Wang K, Huang X-J. 2019. Myeloid-derived suppressor cells in hematological malignancies: friends or foes. J. Hematol. Oncol 12:105 31640764 10.1186/s13045-019-0797-3 PMC6805310

[11] Bussard KM, Mutkus L, Stumpf K, Gomez-Manzano C, Marini FC. 2016. Tumor-associated stromal cells as key contributors to the tumor microenvironment. Breast Cancer Res. 18:84 27515302 10.1186/s13058-016-0740-2 PMC4982339

[12] Jin M-Z, Jin W-L. 2020. The updated landscape of tumor microenvironment and drug repurposing. Signal Transduct. Targeted Ther 5:166 10.1038/s41392-020-00280-x PMC7447642 32843638

[13] Petrova V, Annicchiarico-Petruzzelli M, Melino G, Amelio I. 2018. The hypoxic tumour microenvironment. Oncogenesis 7:10 29362402 10.1038/s41389-017-0011-9 PMC5833859

[14] Qiu GZ, Jin MZ, Dai JX, Sun W, Feng JH, Jin WL. 2017. Reprogramming of the tumor in the hypoxic niche: the emerging concept and associated therapeutic strategies. Trends Pharmacol. Sci 38:669–86 28602395 10.1016/j.tips.2017.05.002

[15] Winkler J, Abisoye-Ogunniyan A, Metcalf KJ, Werb Z. 2020. Concepts of extracellular matrix remodelling in tumour progression and metastasis. Nat. Commun 11:5120 33037194 10.1038/s41467-020-18794-x PMC7547708

[16] Quail DF, Joyce JA. 2013. Microenvironmental regulation of tumor progression and metastasis. Nat. Med 19:1423–37 24202395 10.1038/nm.3394 PMC3954707

[17] Watnick RS. 2012. The role of the tumor microenvironment in regulating angiogenesis. Cold Spring Harb. Perspect. Med 2:a006676 23209177 10.1101/cshperspect.a006676 PMC3543072

[18] Acharyya S, Matrisian L, Welch DR, Massagué J. 2015. Invasion and metastasis. In The Molecular Basis of Cancer, ed. Mendelsohn J, Gray JW, Howley PM, Israel MA, Thompson CB, pp. 269–84.e2. Philadelphia: W.B. Saunders. 4th ed.

[19] Walker C, Mojares E, Del Río Hernández A. 2018. Role of extracellular matrix in development and cancer progression. Int. J. Mol. Sci 19:3028 30287763 10.3390/ijms19103028 PMC6213383

[20] Chiang SP, Cabrera RM, Segall JE. 2016. Tumor cell intravasation. Am. J. Physiol. Cell Physiol 311:C1–14 27076614 10.1152/ajpcell.00238.2015 PMC4967137

[21] Sznurkowska MK, Aceto N. 2022. The gate to metastasis: key players in cancer cell intravasation. FEBS J. 289:4336–54 34077633 10.1111/febs.16046 PMC9546053

[22] Wyckoff JB, Wang Y, Lin EY, Li J-F, Goswami S, 2007. Direct visualization of macrophage-assisted tumor cell intravasation in mammary tumors. Cancer Res. 67:2649–56 17363585 10.1158/0008-5472.CAN-06-1823

[23] Harney AS, Arwert EN, Entenberg D, Wang Y, Guo P, 2015. Real-time imaging reveals local, transient vascular permeability, and tumor cell intravasation stimulated by TIE2^hi^ macrophage–derived VEGFA. Cancer Discov. 5:932–43 26269515 10.1158/2159-8290.CD-15-0012 PMC4560669

[24] Welch DR, Hurst DR. 2019. Defining the hallmarks of metastasis. Cancer Res. 79:3011–27 31053634 10.1158/0008-5472.CAN-19-0458 PMC6571042

[25] Roh-Johnson M, Bravo-Cordero JJ, Patsialou A, Sharma VP, Guo P, 2014. Macrophage contact induces RhoA GTPase signaling to trigger tumor cell intravasation. Oncogene 33:4203–12 24056963 10.1038/onc.2013.377 PMC3962803

[26] Lin Y, Xu J, Lan H. 2019. Tumor-associated macrophages in tumor metastasis: biological roles and clinical therapeutic applications. J. Hematol. Oncol 12:76 31300030 10.1186/s13045-019-0760-3 PMC6626377

[27] Massagué J, Obenauf AC. 2016. Metastatic colonization by circulating tumour cells. Nature 529:298–306 26791720 10.1038/nature17038 PMC5029466

[28] Kang DS, Moriarty A, Oh JM, Begum HM, Shen K, Yu M. 2023. Biophysical properties and isolation of circulating tumor cells. In Engineering and Physical Approaches to Cancer, ed. Wong IY, Dawson MR, pp. 255–83. Cham: Springer Int. Publ.

[29] Aceto N, Bardia A, Miyamoto DT, Donaldson MC, Wittner BS, 2014. Circulating tumor cell clusters are oligoclonal precursors of breast cancer metastasis. Cell 158:1110–22 25171411 10.1016/j.cell.2014.07.013 PMC4149753

[30] Yu M, Bardia A, Wittner BS, Stott SL, Smas ME, 2013. Circulating breast tumor cells exhibit dynamic changes in epithelial and mesenchymal composition. Science 339:580–84 23372014 10.1126/science.1228522 PMC3760262

[31] Leach J, Morton JP, Sansom OJ. 2019. Neutrophils: homing in on the myeloid mechanisms of metastasis. Mol. Immunol 110:69–76 29269005 10.1016/j.molimm.2017.12.013 PMC6544568

[32] Gay LJ, Felding-Habermann B. 2011. Contribution of platelets to tumour metastasis. Nat. Rev. Cancer 11:123–34 21258396 10.1038/nrc3004 PMC6894505

[33] Gkountela S, Castro-Giner F, Szczerba BM, Vetter M, Landin J, 2019. Circulating tumor cell clustering shapes DNA methylation to enable metastasis seeding. Cell 176:98–112.e14 30633912 10.1016/j.cell.2018.11.046 PMC6363966

[34] Hamidi H, Ivaska J. 2018. Every step of the way: integrins in cancer progression and metastasis. Nat. Rev. Cancer 18:533–48 30002479 10.1038/s41568-018-0038-z PMC6629548

[35] Hill CN, Hernández-Cáceres MP, Asencio C, Torres B, Solis B, Owen GI. 2020. Deciphering the role of the coagulation cascade and autophagy in cancer-related thrombosis and metastasis. Front. Oncol 10:605314 33365273 10.3389/fonc.2020.605314 PMC7750537

[36] Reymond N, d’Água BB, Ridley AJ. 2013. Crossing the endothelial barrier during metastasis. Nat. Rev. Cancer 13:858–70 24263189 10.1038/nrc3628

[37] Strilic B, Yang L, Albarrán-Juárez J, Wachsmuth L, Han K, 2016. Tumour-cell-induced endothelial cell necroptosis via death receptor 6 promotes metastasis. Nature 536:215–18 27487218 10.1038/nature19076

[38] Braun S, Vogl FD, Naume B, Janni W, Osborne MP, 2005. A pooled analysis of bone marrow micrometastasis in breast cancer. N. Engl. J. Med 353:793–802 16120859 10.1056/NEJMoa050434

[39] Wang C, Luo D. 2021. The metabolic adaptation mechanism of metastatic organotropism. Exp. Hematol. Oncol 10:30 33926551 10.1186/s40164-021-00223-4 PMC8082854

[40] Azubuike UF, Tanner K. 2023. Biophysical determinants of cancer organotropism. Trends Cancer 9:188–97 36494310 10.1016/j.trecan.2022.11.002

[41] Chen W, Hoffmann AD, Liu H, Liu X. 2018. Organotropism: new insights into molecular mechanisms of breast cancer metastasis. NPJ Precision Oncol. 2:4 10.1038/s41698-018-0047-0 PMC5871901 29872722

[42] Aird WC. 2007. Phenotypic heterogeneity of the endothelium. Circ. Res 100:158–73 17272818 10.1161/01.RES.0000255691.76142.4a

[43] Croucher PI, McDonald MM, Martin TJ. 2016. Bone metastasis: the importance of the neighbourhood. Nat. Rev. Cancer 16:373–86 27220481 10.1038/nrc.2016.44

[44] Zarrintaj P, Saeb MR, Stadler FJ, Yazdi MK, Nezhad MN, 2022. Human organs-on-chips: a review of the state-of-the-art, current prospects, and future challenges. Adv. Biol 6:2000526 10.1002/adbi.202000526 34837667

[45] Sackmann EK, Fulton AL, Beebe DJ. 2014. The present and future role of microfluidics in biomedical research. Nature 507:181–89 24622198 10.1038/nature13118

[46] Niculescu AG, Chircov C, Bîrcă AC, Grumezescu AM. 2021. Fabrication and applications of microfluidic devices: a review. Int. J. Mol. Sci 22:2011 33670545 10.3390/ijms22042011 PMC7921936

[47] Folch A 2013. Introduction to BioMEMS. Boca Raton, FL: CRC Press

[48] Begum HM, Ta HP, Zhou H, Ando Y, Kang D, 2019. Spatial regulation of mitochondrial heterogeneity by stromal confinement in micropatterned tumor models. Sci. Rep 9:11187 31371796 10.1038/s41598-019-47593-8 PMC6671984

[49] Oh JM, Begum HM, Liu YL, Ren Y, Shen K. 2022. Recapitulating tumor hypoxia in a cleanroom-free, liquid-pinning-based microfluidic tumor model. ACS Biomater. Sci. Eng 8:3107–21 35678715 10.1021/acsbiomaterials.2c00207 PMC9299272

[50] Anderson NM, Simon MC. 2020. The tumor microenvironment. Curr. Biol 30:R921–25 32810447 10.1016/j.cub.2020.06.081 PMC8194051

[51] Agrawal B 2019. New therapeutic targets for cancer: the interplay between immune and metabolic checkpoints and gut microbiota. Clin. Transl. Med 8:e23 10.1186/s40169-019-0241-x PMC6715761 31468283

[52] de Visser KE, Joyce JA. 2023. The evolving tumor microenvironment: from cancer initiation to metastatic outgrowth. Cancer Cell 41:374–403 36917948 10.1016/j.ccell.2023.02.016

[53] Man SM, Jenkins BJ. 2022. Context-dependent functions of pattern recognition receptors in cancer. Nat. Rev. Cancer 22:397–413 35355007 10.1038/s41568-022-00462-5

[54] Mason J, Öhlund D. 2023. Key aspects for conception and construction of co-culture models of tumor-stroma interactions. Front. Bioeng. Biotechnol 11:1150764 37091337 10.3389/fbioe.2023.1150764 PMC10119418

[55] Kim H, Schaniel C. 2018. Modeling hematological diseases and cancer with patient-specific induced pluripotent stem cells. Front. Immunol 9:2243 30323816 10.3389/fimmu.2018.02243 PMC6172418

[56] Li C, Holman JB, Shi Z, Qiu B, Ding W. 2023. On-chip modeling of tumor evolution: advances, challenges and opportunities. Mater. Today Bio 21:100724 10.1016/j.mtbio.2023.100724 PMC10359640 37483380

[57] Ronaldson-Bouchard K, Vunjak-Novakovic G. 2018. Organs-on-a-chip: a fast track for engineered human tissues in drug development. Cell Stem Cell 22(3):310–24 29499151 10.1016/j.stem.2018.02.011 PMC5837068

[58] Bonnans C, Chou J, Werb Z. 2014. Remodelling the extracellular matrix in development and disease. Nat. Rev. Mol. Cell Biol 15:786–801 25415508 10.1038/nrm3904 PMC4316204

[59] Langhans SA. 2018. Three-dimensional in vitro cell culture models in drug discovery and drug repositioning. Front. Pharmacol 9:6 29410625 10.3389/fphar.2018.00006 PMC5787088

[60] Henke E, Nandigama R, Ergün S. 2020. Extracellular matrix in the tumor microenvironment and its impact on cancer therapy. Front. Mol. Biosci 6:160 32118030 10.3389/fmolb.2019.00160 PMC7025524

[61] Huang J, Zhang L, Wan D, Zhou L, Zheng S, 2021. Extracellular matrix and its therapeutic potential for cancer treatment. Signal Transduct. Targeted Ther 6:153 10.1038/s41392-021-00544-0 PMC8062524 33888679

[62] Rodrigues J, Heinrich MA, Teixeira LM, Prakash J. 2021. 3D in vitro model (r)evolution: unveiling tumor–stroma interactions. Trends Cancer 7:249–64 33218948 10.1016/j.trecan.2020.10.009

[63] Gil JF, Moura CS, Silverio V, Gonçalves G, Santos HA. 2023. Cancer models on chip: paving the way to large-scale trial applications. Adv. Mater 35:e2300692 37103886 10.1002/adma.202300692

[64] Elango J, Zamora-Ledezma C, Maté-Sánchez de Val JE. 2023. Natural versus synthetic polymers: How do they communicate with cells for skin regeneration—a review. J. Compos. Sci 7:385

[65] Aazmi A, Zhang D, Mazzaglia C, Yu M, Wang Z, 2024. Biofabrication methods for reconstructing extracellular matrix mimetics. Bioactive Mater. 31:475–96 10.1016/j.bioactmat.2023.08.018 PMC10500422 37719085

[66] De Wever O, Pauwels P, De Craene B, Sabbah M, Emami S, 2008. Molecular and pathological signatures of epithelial–mesenchymal transitions at the cancer invasion front. Histochem. Cell Biol 130:481–94 18648847 10.1007/s00418-008-0464-1 PMC2522326

[67] Shen K, Luk S, Hicks DF, Elman JS, Bohr S, 2014. Resolving cancer–stroma interfacial signalling and interventions with micropatterned tumour–stromal assays. Nat. Commun 5:5662 25489927 10.1038/ncomms6662 PMC4261930

[68] Ahmed MAM, Nagelkerke A. 2021. Current developments in modelling the tumour microenvironment in vitro: incorporation of biochemical and physical gradients. Organs-on-a-Chip 3:100012

[69] Wang HF, Ran R, Liu Y, Hui Y, Zeng B, 2018. Tumor-vasculature-on-a-chip for investigating nanoparticle extravasation and tumor accumulation. ACS Nano 12:11600–9 30380832 10.1021/acsnano.8b06846

[70] Luo Z, Zhou X, Mandal K, He N, Wennerberg W, 2021. Reconstructing the tumor architecture into organoids. Adv. Drug Deliv. Rev 176:113839 34153370 10.1016/j.addr.2021.113839 PMC8560135

[71] Nia HT, Munn LL, Jain RK. 2020. Physical traits of cancer. Science 370(6516):eaaz0868 33122355 10.1126/science.aaz0868 PMC8274378

[72] Stylianopoulos T, Martin JD, Chauhan VP, Jain SR, Diop-Frimpong B, 2012. Causes, consequences, and remedies for growth-induced solid stress in murine and human tumors. PNAS 109:15101–8 22932871 10.1073/pnas.1213353109 PMC3458380

[73] Heldin C-H, Rubin K, Pietras K, Östman A. 2004. High interstitial fluid pressure—an obstacle in cancer therapy. Nat. Rev. Cancer 4:806–13 15510161 10.1038/nrc1456

[74] Begum HM, Oh JM, Kang DS, Yu M, Shen K. 2023. Physical regulations of cell interactions and metabolism in tumor microenvironments. In Engineering and Physical Approaches to Cancer, ed. Wong IY, Dawson MR, pp. 139–57. Cham: Springer Int. Publ.

[75] Yan J, Chen Y, Luo M, Hu X, Li H, 2023. Chronic stress in solid tumor development: from mechanisms to interventions. J. Biomed. Sci 30:8 36707854 10.1186/s12929-023-00903-9 PMC9883141

[76] Martín-Asensio A, Dávila S, Cacheux J, Lindstaedt A, Dziadosz A, 2023. Recapitulating solid stress on tumor on a chip for nanomedicine diffusive transport prediction. Adv. NanoBiomed Res 3:2200164

[77] Hassell BA, Goyal G, Lee E, Sontheimer-Phelps A, Levy O, 2017. Human organ chip models recapitulate orthotopic lung cancer growth, therapeutic responses, and tumor dormancy in vitro. Cell Rep. 21:508–16 29020635 10.1016/j.celrep.2017.09.043

[78] Xiao Y, Kim D, Dura B, Zhang K, Yan R, 2019. Ex vivo dynamics of human glioblastoma cells in a microvasculature-on-a-chip system correlates with tumor heterogeneity and subtypes. Adv. Sci 6:1801531 10.1002/advs.201801531 PMC6468969 31016107

[79] Halldorsson S, Lucumi E, Gómez-Sjöberg R, Fleming RMT. 2015. Advantages and challenges of microfluidic cell culture in polydimethylsiloxane devices. Biosens. Bioelectron 63:218–31 25105943 10.1016/j.bios.2014.07.029

[80] Schneider G, Schmidt-Supprian M, Rad R, Saur D. 2017. Tissue-specific tumorigenesis: context matters. Nat. Rev. Cancer 17:239–53 28256574 10.1038/nrc.2017.5 PMC5823237

[81] Krausgruber T, Fortelny N, Fife-Gernedl V, Senekowitsch M, Schuster LC, 2020. Structural cells are key regulators of organ-specific immune responses. Nature 583:296–302 32612232 10.1038/s41586-020-2424-4 PMC7610345

[82] Marcu R, Choi YJ, Xue J, Fortin CL, Wang Y, 2018. Human organ-specific endothelial cell heterogeneity. iScience 4:20–35 30240741 10.1016/j.isci.2018.05.003 PMC6147238

[83] Deasy SK, Erez N. 2022. A glitch in the matrix: organ-specific matrisomes in metastatic niches. Trends Cell Biol. 32:110–23 34479765 10.1016/j.tcb.2021.08.001

[84] Lee CZW, Ginhoux F. 2022. Biology of resident tissue macrophages. Development 149:dev200270 10.1242/dev.200270 35502781

[85] Schenkel JM, Masopust D. 2014. Tissue-resident memory T cells. Immunity 41:886–97 25526304 10.1016/j.immuni.2014.12.007 PMC4276131

[86] Siegel RL, Miller KD, Wagle NS, Jemal A. 2023. Cancer statistics, 2023. CA Cancer J. Clin 73:17–48 36633525 10.3322/caac.21763

[87] Paek J, Park SE, Lu Q, Park K-T, Cho M, 2019. Microphysiological engineering of self-assembled and perfusable microvascular beds for the production of vascularized three-dimensional human microtissues. ACS Nano 13:7627–43 31194909 10.1021/acsnano.9b00686

[88] Liu W, Song J, Du X, Zhou Y, Li Y, 2019. AKR1B10 (aldo-keto reductase family 1 B10) promotes brain metastasis of lung cancer cells in a multi-organ microfluidic chip model. Acta Biomater. 91:195–208 31034948 10.1016/j.actbio.2019.04.053

[89] Kim Y, Ko J, Shin N, Park S, Lee S-R, 2022. All-in-one microfluidic design to integrate vascularized tumor spheroid into high-throughput platform. Biotechnol. Bioeng 119:3678–93 36043394 10.1002/bit.28221

[90] Shen P, Jia Y, Zhou W, Zheng W, Wu Y, 2023. A biomimetic liver cancer on-a-chip reveals a critical role of LIPOCALIN-2 in promoting hepatocellular carcinoma progression. Acta Pharm. Sin. B 13:4621–37 37969730 10.1016/j.apsb.2023.04.010 PMC10638501

[91] Nuciforo S, Fofana I, Matter MS, Blumer T, Calabrese D, 2018. Organoid models of human liver cancers derived from tumor needle biopsies. Cell Rep. 24:1363–76 30067989 10.1016/j.celrep.2018.07.001 PMC6088153

[92] Baek S, Ha H-S, Park JS, Cho MJ, Kim H-S, 2024. Chip collection of hepatocellular carcinoma based on O_2_ heterogeneity from patient tissue. Nat. Commun 15:5117 38879551 10.1038/s41467-024-49386-8 PMC11180182

[93] Miller AJ, Spence JR. 2017. In vitro models to study human lung development, disease and homeostasis. Physiology 32:246–60 28404740 10.1152/physiol.00041.2016 PMC6148341

[94] Del Piccolo N, Shirure VS, Bi Y, Goedegebuure SP, Gholami S, 2021. Tumor-on-chip modeling of organ-specific cancer and metastasis. Adv. Drug Deliv. Rev 175:113798 34015419 10.1016/j.addr.2021.05.008

[95] Waters CM, Roan E, Navajas D. 2012. Mechanobiology in lung epithelial cells: measurements, perturbations, and responses. Compr. Physiol 2:1–29 23728969 10.1002/cphy.c100090 PMC4457445

[96] Mirhadi S, Tam S, Li Q, Moghal N, Pham N-A, 2022. Integrative analysis of non-small cell lung cancer patient-derived xenografts identifies distinct proteotypes associated with patient outcomes. Nat. Commun 13:1811 35383171 10.1038/s41467-022-29444-9 PMC8983714

[97] Nicholson AG, Tsao MS, Beasley MB, Borczuk AC, Brambilla E, 2022. The 2021 WHO Classification of Lung Tumors: impact of advances since 2015. J. Thorac. Oncol 17:362–87 34808341 10.1016/j.jtho.2021.11.003

[98] Herbst RS, Heymach JV, Lippman SM. 2008. Lung cancer. N. Engl. J. Med 359:1367–80 18815398 10.1056/NEJMra0802714 PMC10662965

[99] Larsen JE, Minna JD. 2011. Molecular biology of lung cancer: clinical implications. Clin. Chest Med 32:703–40 22054881 10.1016/j.ccm.2011.08.003 PMC3367865

[100] Altorki NK, Markowitz GJ, Gao D, Port JL, Saxena A, 2019. The lung microenvironment: an important regulator of tumour growth and metastasis. Nat. Rev. Cancer 19:9–31 30532012 10.1038/s41568-018-0081-9 PMC6749995

[101] Alexandre J, Hu Y, Lu W, Pelicano H, Huang P. 2007. Novel action of paclitaxel against cancer cells: bystander effect mediated by reactive oxygen species. Cancer Res. 67:3512–17 17440056 10.1158/0008-5472.CAN-06-3914

[102] Trefts E, Gannon M, Wasserman DH. 2017. The liver. Curr. Biol 27:R1147–51 29112863 10.1016/j.cub.2017.09.019 PMC5897118

[103] Ben-Moshe S, Itzkovitz S. 2019. Spatial heterogeneity in the mammalian liver. Nat. Rev. Gastroenterol. Hepatol 16:395–410 30936469 10.1038/s41575-019-0134-x

[104] Grisham JW. 2009. Organizational principles of the liver. In The Liver: Biology and Pathobiology, 5th ed., ed. Arias IM, pp. 1–15, Hoboken, NJ: Wiley

[105] Farazi PA, DePinho RA. 2006. Hepatocellular carcinoma pathogenesis: from genes to environment. Nat. Rev. Cancer 6:674–87 16929323 10.1038/nrc1934

[106] Llovet JM, Kelley RK, Villanueva A, Singal AG, Pikarsky E, 2021. Hepatocellular carcinoma. Nat. Rev. Dis. Primers 7:6 33479224 10.1038/s41572-020-00240-3 PMC13537433

[107] Marrero JA, Kulik LM, Sirlin CB, Zhu AX, Finn RS, 2018. Diagnosis, staging, and management of hepatocellular carcinoma: 2018 practice guidance by the American Association for the Study of Liver Diseases. Hepatology 68:723–50 29624699 10.1002/hep.29913

[108] Barry AE, Baldeosingh R, Lamm R, Patel K, Zhang K, 2020. Hepatic stellate cells and hepatocarcinogenesis. Front. Cell Dev. Biol 8:709 32850829 10.3389/fcell.2020.00709 PMC7419619

[109] Adjei-Sowah EA, O’Connor SA, Veldhuizen J, Lo Cascio C, Plaisier C, 2022. Investigating the interactions of glioma stem cells in the perivascular niche at single-cell resolution using a microfluidic tumor microenvironment model. Adv. Sci 9:2201436 10.1002/advs.202201436 PMC9313491 35619544

[110] Seo S, Nah S-Y, Lee K, Choi N, Kim HN. 2022. Triculture model of in vitro BBB and its application to study BBB-associated chemosensitivity and drug delivery in glioblastoma. Adv. Funct. Mater 32:2106860

[111] Ozturk MS, Lee VK, Zou H, Friedel RH, Intes X, Dai G. 2020. High-resolution tomographic analysis of in vitro 3D glioblastoma tumor model under long-term drug treatment. Sci. Adv 6:eaay7513 32181351 10.1126/sciadv.aay7513 PMC7060061

[112] Komez A, Buyuksungur A, Antmen E, Swieszkowski W, Hasirci N, Hasirci V. 2020. A two-compartment bone tumor model to investigate interactions between healthy and tumor cells. Biomed. Mater 15:035007 31935707 10.1088/1748-605X/ab6b31

[113] Paindelli C, Navone N, Logothetis CJ, Friedl P, Dondossola E. 2019. Engineered bone for probing organotypic growth and therapy response of prostate cancer tumoroids in vitro. Biomaterials 197:296–304 30682644 10.1016/j.biomaterials.2019.01.027 PMC7094882

[114] Bock N, Kryza T, Shokoohmand A, Rohl J, Ravichandran A, 2021. In vitro engineering of a bone metastases model allows for study of the effects of antiandrogen therapies in advanced prostate cancer. Sci. Adv 7:eabg2564 34193425 10.1126/sciadv.abg2564 PMC8245033

[115] Villasante A, Marturano-Kruik A, Robinson ST, Liu Z, Guo XE, Vunjak-Novakovic G. 2017. Tissue-engineered model of human osteolytic bone tumor. Tissue Eng. Part C Methods 23:98–107 28068876 10.1089/ten.tec.2016.0371 PMC5314970

[116] Marturano-Kruik A, Nava MM, Yeager K, Chramiec A, Hao L, 2018. Human bone perivascular niche-on-a-chip for studying metastatic colonization. PNAS 115:1256–61 29363599 10.1073/pnas.1714282115 PMC5819403

[117] Stiles J, Jernigan TL. 2010. The basics of brain development. Neuropsychol. Rev 20:327–48 21042938 10.1007/s11065-010-9148-4 PMC2989000

[118] von Bartheld CS. 2018. Myths and truths about the cellular composition of the human brain: a review of influential concepts. J. Chem. Neuroanat 93:2–15 28873338 10.1016/j.jchemneu.2017.08.004 PMC5834348

[119] Allen NJ, Lyons DA. 2018. Glia as architects of central nervous system formation and function. Science 362:181–85 30309945 10.1126/science.aat0473 PMC6292669

[120] Hajal C, Roi BL, Kamm RD, Maoz BM. 2021. Biology and models of the blood–brain barrier. Annu. Rev. Biomed. Eng 23:359–84 34255993 10.1146/annurev-bioeng-082120-042814

[121] Reinhard J, Brösicke N, Theocharidis U, Faissner A. 2016. The extracellular matrix niche microenvironment of neural and cancer stem cells in the brain. Int. J. Biochem. Cell Biol 81:174–83 27157088 10.1016/j.biocel.2016.05.002

[122] Venkatesh Humsa S, Johung Tessa B, Caretti V, Noll A, Tang Y, 2015. Neuronal activity promotes glioma growth through neuroligin-3 secretion. Cell 161:803–16 25913192 10.1016/j.cell.2015.04.012 PMC4447122

[123] Steeg PS. 2021. The blood–tumour barrier in cancer biology and therapy. Nat. Rev. Clin. Oncol 18:696–714 34253912 10.1038/s41571-021-00529-6

[124] Bao X, Wu J, Xie Y, Kim S, Michelhaugh S, 2020. Protein expression and functional relevance of efflux and uptake drug transporters at the blood–brain barrier of human brain and glioblastoma. Clin. Pharmacol. Ther 107:1116–27 31664714 10.1002/cpt.1710 PMC7167337

[125] Quail DF, Joyce JA. 2017. The microenvironmental landscape of brain tumors. Cancer Cell 31:326–41 28292436 10.1016/j.ccell.2017.02.009 PMC5424263

[126] Tao W, Zhang A, Zhai K, Huang Z, Huang H, 2020. SATB2 drives glioblastoma growth by recruiting CBP to promote FOXM1 expression in glioma stem cells. EMBO Mol. Med 12:e12291 33124191 10.15252/emmm.202012291 PMC7721366

[127] Cornelison RC, Yuan JX, Tate KM, Petrosky A, Beeghly GF, 2022. A patient-designed tissue-engineered model of the infiltrative glioblastoma microenvironment. NPJ Precision Oncol. 6:54 10.1038/s41698-022-00290-8 PMC9338058 35906273

[128] Tang M, Xie Q, Gimple RC, Zhong Z, Tam T, 2020. Three-dimensional bioprinted glioblastoma microenvironments model cellular dependencies and immune interactions. Cell Res. 30:833–53 32499560 10.1038/s41422-020-0338-1 PMC7608409

[129] Florencio-Silva R, Sasso GR, Sasso-Cerri E, Simões MJ, Cerri PS. 2015. Biology of bone tissue: structure, function, and factors that influence bone cells. BioMed. Res. Int 2015:421746 26247020 10.1155/2015/421746 PMC4515490

[130] Clarke B 2008. Normal bone anatomy and physiology. Clin. J. Am. Soc. Nephrol 3(Suppl. 3):S131–39 18988698 10.2215/CJN.04151206 PMC3152283

[131] Mercier FE, Ragu C, Scadden DT. 2012. The bone marrow at the crossroads of blood and immunity. Nat. Rev. Immunol 12:49–60 10.1038/nri3132 PMC4013788 22193770

[132] Morrison SJ, Scadden DT. 2014. The bone marrow niche for haematopoietic stem cells. Nature 505:327–34 24429631 10.1038/nature12984 PMC4514480

[133] Ascenzi M-G, Roe AK. 2012. The osteon: the micromechanical unit of compact bone. Front. Biosci 17:1551–81 10.2741/4003 22201820

[134] Liu Y, Luo D, Wang T. 2016. Hierarchical structures of bone and bioinspired bone tissue engineering. Small 12:4611–32 27322951 10.1002/smll.201600626

[135] Chai YC, Carlier A, Bolander J, Roberts SJ, Geris L, 2012. Current views on calcium phosphate osteogenicity and the translation into effective bone regeneration strategies. Acta Biomater. 8:3876–87 22796326 10.1016/j.actbio.2012.07.002

[136] Palmer LC, Newcomb CJ, Kaltz SR, Spoerke ED, Stupp SI. 2008. Biomimetic systems for hydroxyapatite mineralization inspired by bone and enamel. Chem. Rev 108:4754–83 19006400 10.1021/cr8004422 PMC2593885

[137] Eriksen EF. 2010. Cellular mechanisms of bone remodeling. Rev. Endocr. Metab. Disord 11:219–27 21188536 10.1007/s11154-010-9153-1 PMC3028072

[138] Beird HC, Bielack SS, Flanagan AM, Gill J, Heymann D, 2022. Osteosarcoma. Nat. Rev. Dis. Primers 8:77 36481668 10.1038/s41572-022-00409-y

[139] Odri GA, Tchicaya-Bouanga J, Yoon DJY, Modrowski D. 2022. Metastatic progression of osteosarcomas: a review of current knowledge of environmental versus oncogenic drivers. Cancers 14:360 35053522 10.3390/cancers14020360 PMC8774233

[140] Lee J, Cuddihy MJ, Kotov NA. 2008. Three-dimensional cell culture matrices: state of the art. Tissue Eng. Part B Rev 14:61–86 18454635 10.1089/teb.2007.0150

[141] Offeddu GS, Hajal C, Foley CR, Wan Z, Ibrahim L, 2021. The cancer glycocalyx mediates intravascular adhesion and extravasation during metastatic dissemination. Commun. Biol 4:255 33637851 10.1038/s42003-021-01774-2 PMC7910477

[142] Ghajar CM, Peinado H, Mori H, Matei IR, Evason KJ, 2013. The perivascular niche regulates breast tumour dormancy. Nat. Cell Biol 15:807–17 23728425 10.1038/ncb2767 PMC3826912

[143] Fairfield H, Falank C, Farrell M, Vary C, Boucher JM, 2019. Development of a 3D bone marrow adipose tissue model. Bone 118:77–88 29366838 10.1016/j.bone.2018.01.023 PMC6062483

[144] Chou DB, Frismantas V, Milton Y, David R, Pop-Damkov P, 2020. On-chip recapitulation of clinical bone marrow toxicities and patient-specific pathophysiology. Nat. Biomed. Eng 4:394–406 31988457 10.1038/s41551-019-0495-z PMC7160021

[145] Glaser DE, Curtis MB, Sariano PA, Rollins ZA, Shergill BS, 2022. Organ-on-a-chip model of vascularized human bone marrow niches. Biomaterials 280:121245 34810038 10.1016/j.biomaterials.2021.121245 PMC10658812

[146] Ma C, Witkowski MT, Harris J, Dolgalev I, Sreeram S, 2020. Leukemia-on-a-chip: dissecting the chemoresistance mechanisms in B cell acute lymphoblastic leukemia bone marrow niche. Sci. Adv 6:eaba5536 33127669 10.1126/sciadv.aba5536 PMC7608809

[147] Gonzalez Diaz EC, Tai M, Monette CEF, Wu JY, Yang F. 2023. Spatially patterned 3D model mimics key features of cancer metastasis to bone. Biomaterials 299:122163 37236137 10.1016/j.biomaterials.2023.122163 PMC10621670

[148] Choudhary S, Ramasundaram P, Dziopa E, Mannion C, Kissin Y, 2018. Human ex vivo 3D bone model recapitulates osteocyte response to metastatic prostate cancer. Sci. Rep 8:17975 30568232 10.1038/s41598-018-36424-x PMC6299475

[149] Park Y, Cheong E, Kwak JG, Carpenter R, Shim JH, Lee J. 2021. Trabecular bone organoid model for studying the regulation of localized bone remodeling. Sci. Adv 7:eabd6495 33523925 10.1126/sciadv.abd6495 PMC7817107

[150] Park Y, Sato T, Lee J. 2023. Functional and analytical recapitulation of osteoclast biology on demineralized bone paper. Nat. Commun 14:8092 38062034 10.1038/s41467-023-44000-9 PMC10703810

[151] Kwak JG, Lee J. 2023. Bone marrow adipocytes contribute to tumor microenvironment-driven chemoresistance via sequestration of doxorubicin. Cancers 15:2737 37345073 10.3390/cancers15102737 PMC10216070

[152] Bonewald LF. 2011. The amazing osteocyte. J. Bone Miner. Res 26:229–38 21254230 10.1002/jbmr.320 PMC3179345

[153] Yoon H, Park Y, Kwak J-G, Lee J. 2024. Collagen structures of demineralized bone paper direct mineral metabolism. JBMR Plus 8:ziae080 38989259 10.1093/jbmrpl/ziae080 PMC11235081

